# Supplementary material for: Home, sweet home? The impact of working from home on the division of unpaid work during the COVID-19 lockdown
Source: PLoS One. 2021 Nov 8;16(11):e0259580. doi: 10.1371/journal.pone.0259580 (PMC8575241; doi:10.1371/journal.pone.0259580)
Supplement: S1 File — (PDF) [file pone.0259580.s001.pdf]

S1 Supporting information.
Additional figures, Descriptive statistics, & Robustness tests

S.A Additional figures

Fig. S1. Average agreement with statements on WFH from couple households without children

Fig. S2. Agreement with statements on reconciliation of family and WFH by gender

Fig. S3. Division of unpaid work before and during COVID-19 lockdown

Fig. S1. Average agreement with statements on WFH from couple households without children

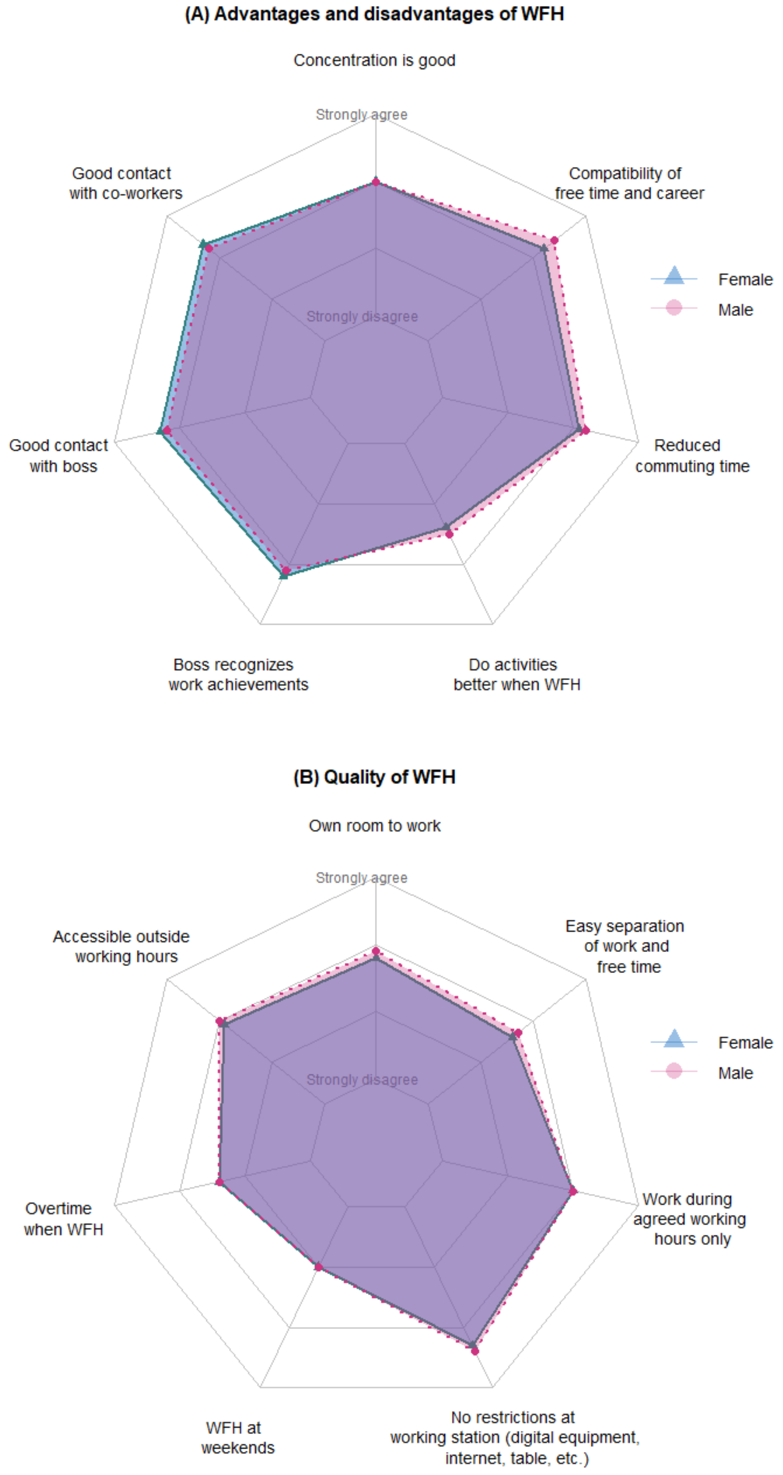

*Reading example:*This radar chart displays the average agreement with different statements on WFH. The smaller the distance on the axis to the centre, the more the respondents disagree with the statement. Blue triangles represent answers from women, pink circles represent men's responses. On average, there is a high degree of similarity in answers to statements of men (pink circles) and women (blue triangles) indicated by the almost overlapping points on the different axis.

Fig. S2. Agreement with statements on reconciliation of family and WFH by gender

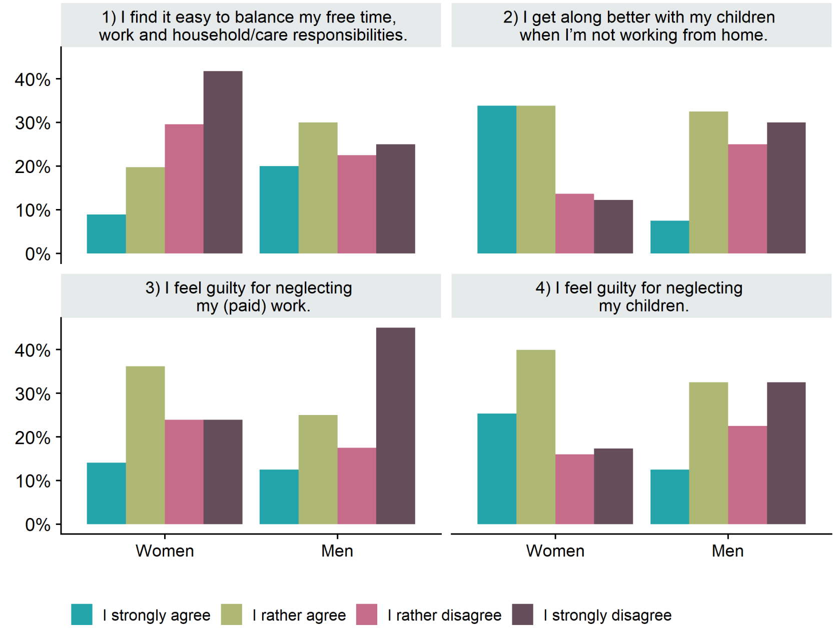

Reading example: This bar chart shows the distribution of agreement (from left to right “Strongly agree” (blue), “Rather agree” (green), “Rather disagree” (pink) to “Strongly disagree” (purple)) for four different statements by parents WFH. Statement 1 indicates for example that 42% of all mothers strongly disagree with “easy reconciliation at home”, whereas only 25% of all fathers strongly disagree with this statement.

Fig. S3. Division of unpaid work before and during COVID-19 lockdown

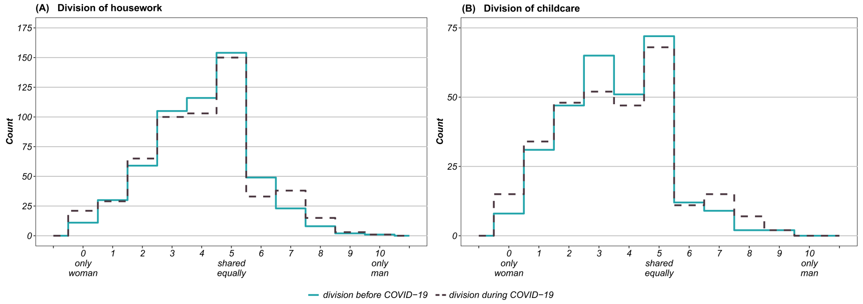

Reading example: The two lines in Fig. S3A and Fig. 3B show the overall distribution of unpaid work before (blue and continuous line) and during (grey-purple dashed line) the lockdown, for housework (S3A) and childcare (S3B) respectively. For scale no. 0 (indicating the “woman does everything”) we see that the number of households reporting this value increased during the lockdown, i.e. almost doubled.

S.B Descriptive statistics

Tab. S1. Survey sample size and key variables

Tab. S2. Division of unpaid work before and during the lockdown

Tab. S3. Average time spent per activity during the lockdown by gender

Table S1. Survey sample size and key variables

Couple and Household Characteristics ($n = 558$)

Change in the division of housework (HW): ♂ does more

	True	*Not true*
n	155	403
%	27.8	72.2

Change in the division of childcare tasks (CC): ♂ does more

	True	*Not True*	*NA*
n	101	198	259
%	18.1	35.5	46.4

Working from home

	Both	*No one*	*Only woman*	*Only man*
n	359	55	102	42
%	64.3	9.9	18.3	7.5

Income situation

	♀ more	*∼ same*	*♂ more*
n	70	237	251
%	12.5	42.5	45.0

Division of housework (HW) before lockdown

	♀ much more	*♀ more*	*equal*	*♂ more*	*♂ much more*
n	100	221	154	72	11
%	17.9	39.6	27.6	12.9	2.0

Division of childcare (CC) before lockdown

	♀ much more	*♀ more*	*equal*	*♂ more*	*♂ much more*	*NA*
n	86	116	72	21	4	259
%	15.4	20.8	12.9	3.8	0.7	46.4

Household type

	Couple w/o children <15	*Couple with children <15*	*Other*
n	235	277	46
%	42.1	49.6	8.2

Average number of children by age group

	0 – 2 years	*3 – 5 years*	*6 – 9 years*	*10 – 14 years*
∅	0.14	0.24	0.25	0.21

Region

	Burgen-land	*Carinthia*	*Lower Austria*	*Upper Austria*	*Salzburg*	*Styria*	*Tyrol*	*Vor-arlberg*	*Vienna*	*Other*
n	24	16	91	51	20	41	11	5	277	22
%	4.3	2.9	16.3	9.1	3.6	7.3	2.0	0.9	49.6	3.9

Individual Characteristics ($n = 1116$)

Average age

	♀	♂
∅	40.8	43.5

Highest education completed

	Primary/ lower second.		*Higher second.*		*Tertiary*	
	♀	♂	♀	♂	♀	♂
n	73	133	88	123	397	302
%	13.1	23.8	15.8	22.0	71.1	54.1

Employment status

	Employed		*Self-employed*		*Short-time work*	
	♀	♂	♀	♂	♀	♂
n	474	442	46	69	38	47
%	84.9	79.2	8.2	12.4	6.8	8.4

Working hours: part-time (⩽20h)

	Full-time		*Full-time (ST)*		*Part-time*		*Part-time (ST)*	
	♀	♂	♀	♂	♀	♂	♀	♂
n	402	482	4	17	118	29	34	30
%	72.0	86.4	0.7	3.0	21.1	5.2	6.1	5.4

Note: ST=short-time; NA=not available

Table S2. Division of unpaid work before and during the lockdown

	♀ *does everything*	*1*	*2*	*3*	*4*	*5*	*6*	*7*	*8*	*9*	♂ *does everything*	*NA*
**Distribution of housework (HW) before lockdown**												
n	11	30	59	105	116	154	49	23	8	2	1	
%	2.0	5.4	10.6	18.8	20.8	27.6	8.8	4.1	1.4	0.4	0.2	
**Distribution of childcare (CC) before lockdown**												
n	8	31	47	65	51	72	12	9	2	2	0	259
%	1.4	5.6	8.4	11.6	9.1	12.9	2.2	1.6	0.4	0.4	0	46.4
**Distribution of housework (HW) during lockdown**												
n	21	29	65	100	103	150	33	38	15	3	1	
%	3.8	5.2	11.6	17.9	18.5	26.9	5.9	6.8	2.7	0.5	0.2	
**Distribution of childcare (CC) during lockdown**												
n	15	34	48	52	47	68	11	15	7	2	0	259
%	2.7	6.1	8.6	9.3	8.4	12.2	2.0	2.7	1.3	0.4	0	46.4

Note: ST=short-time; NA=not available

Table S3. Average time spent per activity during the lockdown by gender

	Male (N=550)			*Female (N=556)*		
	∅[a]	Participation rate[b]	∅ participants[c]	∅[a]	Participation rate[b]	∅ participants[c]
Activity	hh:mm	%	hh:mm	hh:mm	%	hh:mm
Paid work	07:48	97.6	08:00	06:44	98.7	06:49
Housework						
Cooking, baking, grocery shopping	00:52	75.8	01:09	01:29	95.7	01:33
Cleaning, laundry	00:41	75.5	00:55	01:09	93.5	01:14
Other: pet care, gardening, repairs	00:37	55.5	01:07	00:29	55.9	00:52
Childcare						
Physical care: feeding, washing, supervision	00:39	44.2	01:29	00:55	50.2	01:50
Learning, teaching	00:18	23.6	01:15	00:37	35.3	01:44
Leisure time: reading, playing, speaking with child	00:55	49.3	01:51	01:18	53.4	02:27
Personal care						
Sleeping	07:13	100.0	07:13	07:13	100.0	07:13
Eating, drinking, washing, breaks	01:44	97.5	01:47	01:42	96.8	01:45
Leisure time						
Sports, hobbies, media use	02:00	83.5	02:23	01:13	74.6	01:38
Social contacts	00:46	72.4	01:03	00:46	81.7	00:57
Voluntary work						
Helping high-risk group	00:05	9.8	00:52	00:07	15.5	00:46
Other: Red Cross, etc.	00:03	3.5	01:36	00:02	3.6	00:42
Other activity: Not specified	00:18	20.9	01:27	00:16	21.2	01:14
Total						
Housework	02:11	92.5	02:21	03:07	98.9	03:09
Childcare	01:52	56.7	03:17	02:50	60.3	04:43
Unpaid work	04:03	97.6	04:09	05:58	99.3	06:00
Paid and unpaid work	11:51	100.0	11:50	12:41	100.0	12:41

Note: Estimates by self and by partner are taken into account. If both partners filled out the survey, only the self-reported estimates are used.
[a]Mean time spent by all individuals.
[b]Share of individuals who spent some time on the activity.
[c]Mean time of all individuals who spent some time on the activity.

We asked respondents how they and their partner spent the previous working day during the lockdown, summarized in table S3. The results reveal that women in working couples spent, on average, almost two hours more on unpaid work than men (4h03 compared to 5h58) per day. The average time spent on paid work by women amounts to 6h44 compared to 7h48 for men. It is revealing to look beyond the average time spent on a specific activity by carefully examining differences in the participation rates (i.e. the share of respondents having spent some time on a certain activity) and the average time spent on distinct activities based on this ”participating” subsample. We find that 75% of men have participated in housework activities, such as cooking and cleaning on their previous working day, whereas almost all women have done some housework. Comparing men and women who have participated in housework activities, we observe that these women spent around 25 minutes more on these tasks. For childcare activities, we also note large gender differences. Half of the woman participated in physical childcare (feeding, washing and supervision), and these women spent on average 1h50 on this activity. Among men, both the participation rate (44%) and the average time spent on taking care of children (1h29) was slightly lower. The gender difference is much larger for home-schooling related activities. More than one third of of mothers studied with their children, compared to one forth of fathers, and they spent roughly half an hour more on home-schooling than fathers (1h44 compared to 1h15). The mean time spent on childcare, among those having done any childcare activity, amounts to 3h17 for fathers and 4h43 for mothers. Looking at the time spent on childcare, housework and paid work during the lockdown jointly reveals very long overall working days for parents, in particular for mothers.

S.C Robustness tests

We conduct a series of robustness tests to check whether the results presented in the main text are robust in terms of (S.C.i) the sample definition, (S.C.ii) controlling for whether the questions on time use and characteristics of a partner were answered by a male, (S.C.iii) different definitions of the control variables, and (S.C.iv) modified definitions of the dependent variable. In section (S.C.v) we present the results of models presented in the main text based on a linear probability model, instead of a logistic regression. Note that, as we present average marginal effects, this serves as an indirect validation of the average marginal effects.

Tab. S4. Additional model (4): households without children

Tab. S5. Exclusion of respondents who work partly from home

Tab. S6. Additional control variable: respondent is male

Tab. S7. Definition of the income variable: subjective assessment

Tab. S8. Definition of working hours: continuous working hours

Tab. S9. Definition of working hours: part-time $\leqslant 35h$

Tab. S10. Definition of age variable: age groups

Tab. S11. Definition of children living in the household: age youngest child

Tab. S12. Definition of the dependent variable: woman works more

Tab. S13. Definition of the dependent variable: more equal division of unpaid work within the household

Tab. S14. Linear probability model

S.C.i Sample composition

From the overall sample, we selected 730 heterosexual couples (1,460 adult individuals) in a first step, who are living in the same household, where both partners were either (self-)employed or in short-time work at the point when the survey was answered, and who answered the partner module of the questionnaire or linked their responses via anonymous partner IDs. Due to missing information, mainly in the income variable, the resulting sample corresponding to model (1) of the main text consists of 558 couples. In models (2) and (3), this sample is reduced to the 299 couples with children under 15 years of age.

Model (1), explaining the change in the division of housework, is based on a sample consisting of households with and without children. Model

(4) presented in Table S4 is based on a sample of households without children. Thereby, we can check whether the results presented in the main text are driven by households with or without children. The effect of WFH is insignificant in model (4). Thus, we do not find evidence that WFH influences the probability of men increasing their share of unpaid work in childless couple households. In other words, we do not find evidence that WFH influences the probability of men increasing their share of unpaid work within childless households. This also indicates that the effects of model (1), as presented in the main analysis, and based on households with and without children, are driven by households with children, where either men do more housework if both parents are WFH (but not more childcare), or fathers take on more housework (and childcare) if they alone are WFH.

In an additional robustness check (see Table S5), we excluded individuals who worked only partly, and not entirely, from home, which does not have a significant impact on the results.

S.C.ii Controlling for the gender of the survey respondent

This check concerns the fact that 79.6% of the couple questionnaires were filled out by women. Therefore, we test whether the main results change if we control for the gender of the respondent by including a binary variable which takes the value one if the questionnaire was filled out by the male partner (see Table S6). In fact, this variable is highly significant for housework but not for childcare tasks. Moreover, the probability that men take on more housework is no longer significant for the whole sample. This is, however, no surprise as Table S4 already revealed that this effect is driven by households with children.

S.C.iii Specification of the control variables

The variable defining the relative income of the partners presented in the main text is based on categorical income variables. In Table S7 we employ a variable that is based on a subjective assessment of the income difference between partners. Respondents had to report the perceived difference from their partners (low, equal, high). In this robustness test, we make use of this variable. However, the results are not driven by the definition of the income variable and related measurement errors.

Furthermore, we alter the specification of the working hours variable. In one specification (see Table S8), we use continuous working hours instead of a categorical variable. Although the results for each additional hour worked are highly significant, they are small in magnitude. Thus, the effect of each hour is very small, confirming the results obtained by measuring hours worked for pay in categories. In a similar exercise, we vary the definition of part-time work. In the analysis presented in main text, respondents are classified as working part-time in the event that they worked fewer than 20 hours per week for pay. In the models presented in Table S9, those working fewer than 35 hours are classified as working part time. We find that men who work fewer than 35 hours a week without any short-time work arrangement have a significantly higher probability of

taking on more housework and childcare during lockdown.

Controlling for age by means of age groups instead of a continuous definition (see Table S10), we detect no major changes in the results.

S.C.iv Specification of the dependent variable

We check the possibility that the results are driven by the definition of the dependent variable. Thus, we change the dependent variable to a dummy variable indicating whether the *woman* instead of the *man* within a couple took on more unpaid work during the lockdown. The results are presented in Table S12 and show that the main variable of interest – working from home – is not significant for this specification. Moreover, several other variables having a significant effect on the probability that the male partner within a couple takes on a greater share of unpaid work have no significant effect on the probability that a woman takes on more housework or childcare tasks (such as income and employment status). The only variable that remains highly significant is the pre-lockdown division of unpaid work. We conclude that the unequal division of unpaid work prior to the COVID-19 restrictions and the prevailing gender norms associated with it appear to be the most important predictor.

Furthermore, we changed the dependent variable to a binary variable that becomes one in the event that the division of unpaid work was *more equal*[1] during the COVID-19 restrictions than before (see Table S13). The results show that only the male partner WFH has a positive effect on the probability that the division of unpaid work becomes more equal, even though the effect for the whole sample is no longer significant (as in Table 1). Also it has a positive and significant effect in all three model specifications if both partners are WFH. In the models presented in the main text (Table 1), the effect of both partners WFH on the probability that a man takes on more childcare tasks is also positive but not significant. This could come from the fact that this dependent variable also responds to the case where the male partner took over a larger proportion of the childcare tasks before the lockdown and the woman increased her share during the COVID-19 restrictions (see Fig. 3 in the main text). If the housework or childcare activities had already been equally distributed before the lockdown, it has a (highly significant) negative effect on the probability that unpaid work was even more equally distributed during the COVID-19 restrictions compared to households where the woman previously did much more unpaid work than her male partner. This finding is in line with the main results. The distribution of income within the couple has a positive significant effect on the division of housework in family households if the male partner earns more (similar to the base model), but is not significant for any other model or category. The results for the remaining explanatory variables are similar to the base model, even though some covariates are no longer significant.

S.C.v Linear probability model

The results presented in the main text and the previous robustness tests are based on a logistic regression, estimated by maximum likelihood. In the corresponding tables, we report average marginal effects. In this section, we

estimated the models corresponding to Table 1 based on a linear probability model specification estimated by ordinary least squares. This serves as an indirect test, as the average marginal effects should correspond to the effects of the linear probability model. Table S14 shows that the results do not differ between these model specifications.

Table S4. Additional model (4) households without children

	Dependent variable:			
	more HW: ♂ (1)	more HW: ♂ (2)	more CC: ♂ (3)	more HW: ♂ (4)
WFH: both	0.15 (0.07)**	0.19 (0.10)**	0.11 (0.10)	0.09 (0.11)
WFH: only ♀	0.11 (0.10)	0.06 (0.15)	−0.04 (0.12)	0.17 (0.13)
WFH: only ♂	0.23 (0.12)**	0.42 (0.14)***	0.30 (0.12)**	0.06 (0.16)
WFH: nobody (= *ref*)				
HW before: ♀ more	0.17 (0.05)***	0.15 (0.06)**		0.22 (0.07)***
HW before: ♀ much more	0.32 (0.06)***	0.33 (0.08)***		0.35 (0.09)***
HW before: ♂ (much) more	0.07 (0.07)	0.14 (0.11)		0.06 (0.09)
HW before: equal (= *ref*)				
CC before: ♀ more			0.21 (0.06)***	
CC before: ♀ much more			0.36 (0.07)***	
CC before: ♂ (much) more			0.04 (0.12)	
CC before: equal (= *ref*)				
Higher income: ♀	0.17 (0.07)**	0.21 (0.11)*	−0.01 (0.10)	0.16 (0.09)*
Higher income: ♂	0.09 (0.04)**	0.11 (0.06)**	0.06 (0.06)	0.13 (0.07)**
Equal income (= *ref*)				
Working hours <20h: ♀	0.01 (0.05)	−0.01 (0.06)	−0.11 (0.06)*	−0.01 (0.10)
Working hours <20h (ST): ♀	−0.02 (0.08)	0.00 (0.09)	−0.03 (0.09)	−0.04 (0.13)
Working hours >20h (ST): ♀	−0.14 (0.15)	−0.18 (0.11)*	0.05 (0.22)	
Working hours >20h: ♀ (= *ref*)				
Working hours <20h: ♂	0.15 (0.10)	0.34 (0.13)***	0.46 (0.09)***	−0.03 (0.13)
Working hours <20h (ST): ♂	0.06 (0.09)	0.03 (0.10)	0.14 (0.11)	0.07 (0.15)
Working hours >20h (ST): ♂	0.01 (0.10)	−0.19 (0.09)**	−0.25 (0.10)**	0.07 (0.14)
Working hours >20h: ♂ (= *ref*)				
Self-employed: ♀	−0.06 (0.06)	−0.09 (0.08)	−0.20 (0.07)***	−0.03 (0.10)
Employed: ♀ (= *ref*)				
Self-employed: ♂	−0.07 (0.05)	−0.04 (0.07)	−0.18 (0.07)***	−0.16 (0.08)**
Employed: ♂ (= *ref*)				
No. children 0 – 2 years	−0.05 (0.05)	0.03 (0.07)	0.05 (0.07)	
No. children 3 – 5 years	−0.06 (0.04)	−0.02 (0.05)	0.05 (0.06)	
No. children 6 – 9 years	0.06 (0.03)*	0.09 (0.04)*	0.00 (0.05)	
No. children 10 – 14 years	−0.06 (0.04)	−0.09 (0.06)	−0.17 (0.07)**	
Age: ♀	−0.00 (0.00)	0.01 (0.01)	0.02 (0.01)**	−0.01 (0.01)*
Age: ♂	0.00 (0.00)	0.00 (0.01)	0.00 (0.01)	0.01 (0.01)
Educ. ♀ : Higher sec.	0.02 (0.06)	−0.00 (0.08)	0.09 (0.08)	0.09 (0.08)
Educ. ♀ : Lower sec. | prim.	−0.04 (0.07)	0.01 (0.10)	0.16 (0.11)	−0.03 (0.10)
Educ. ♀ : Tertiary (= *ref*)				
Educ. ♂ : Higher sec.	0.02 (0.05)	0.00 (0.06)	0.06 (0.06)	0.06 (0.07)
Educ. ♂ : Lower sec. | prim.	−0.00 (0.06)	−0.02 (0.09)	−0.09 (0.08)	0.02 (0.08)
Educ. ♂ : Tertiary (= *ref*)				
Observations	558	299	299	259
Log likelihood	−299.55	−148.15	−152.85	−136.79
Deviance	599.09	296.30	305.70	273.58
AIC	653.09	350.30	359.70	317.58
BIC	769.85	450.21	459.61	395.83

$^{***}p < 0.01$; $^{**}p < 0.05$; $^{*}p < 0.1$

Note: WFH=working from home; HW=housework; CC=childcare; ST=short-time

Table S5. Exclusion of respondents who work partly from home

	Dependent variable:		
	more HW: ♂ (1)	more HW: ♂ (2)	more CC: ♂ (3)
WFH: both	0.16 (0.07)**	0.20 (0.10)**	0.11 (0.11)
WFH: only female	0.10 (0.10)	0.05 (0.14)	−0.06 (0.12)
WFH: only ♂	0.25 (0.11)**	0.43 (0.13)***	0.31 (0.12)**
WFH: nobody (= *ref*)			
HW before: ♀ more	0.18 (0.05)***	0.14 (0.06)**	
HW before: ♀ much more	0.30 (0.07)***	0.30 (0.09)***	
HW before: ♂ (much) more	0.06 (0.07)	0.12 (0.10)	
HW before: equal (= *ref*)			
CC before: ♀ more			0.22 (0.07)***
CC before: ♀ much more			0.35 (0.07)***
CC before: ♂ (much) more			0.04 (0.12)
CC before: equal (= *ref*)			
Higher income: ♀	0.19 (0.07)***	0.22 (0.11)**	−0.01 (0.11)
Higher income: ♂	0.07 (0.04)	0.08 (0.06)	0.04 (0.06)
Equal income (= *ref*)			
Working hours ⩽20h: ♀	0.02 (0.05)	0.03 (0.06)	−0.08 (0.06)
Working hours ⩽20h (ST): ♀	0.01 (0.08)	0.06 (0.10)	0.01 (0.10)
Working hours >20h (ST): ♀	−0.15 (0.13)	−0.20 (0.09)**	0.05 (0.23)
Working hours >20h: ♀ (= *ref*)			
Working hours ⩽20h: ♂	0.15 (0.10)	0.35 (0.14)**	0.47 (0.09)***
Working hours ⩽20h (ST): ♂	0.03 (0.09)	−0.00 (0.10)	0.12 (0.11)
Working hours >20h (ST): ♂	−0.03 (0.10)	−0.20 (0.09)**	−0.26 (0.09)***
Working hours >20h: ♂ (= *ref*)			
Self-employed: ♀	−0.07 (0.06)	−0.10 (0.08)	−0.19 (0.08)**
Employed: ♀ (= *ref*)			
Self-employed: ♂	−0.05 (0.06)	−0.03 (0.07)	−0.18 (0.07)***
Employed: ♂ (= *ref*)			
No. children 0 – 2 years	−0.05 (0.06)	0.03 (0.08)	0.06 (0.08)
No. children 3 – 5 years	−0.08 (0.04)*	−0.02 (0.06)	0.06 (0.06)
No. children 6 – 9 years	0.08 (0.04)**	0.11 (0.05)**	0.02 (0.05)
No. children 10 – 14 years	−0.07 (0.04)	−0.08 (0.06)	−0.16 (0.07)**
Age: ♀	−0.00 (0.00)	0.00 (0.01)	0.02 (0.01)**
Age: ♂	0.00 (0.00)	0.01 (0.01)	0.00 (0.01)
Educ. ♀ : Higher sec.	−0.01 (0.05)	−0.02 (0.08)	0.07 (0.09)
Educ. ♀ : Lower sec. | prim.	−0.06 (0.07)	−0.04 (0.10)	0.12 (0.12)
Educ. ♀ : Tertiary (= *ref*)			
Educ. ♂ : Higher sec.	0.05 (0.05)	0.03 (0.07)	0.09 (0.07)
Educ. ♂ : Lower sec. | prim.	0.04 (0.06)	0.06 (0.09)	−0.03 (0.09)
Educ. ♂ : Tertiary (= *ref*)			
Observations	526	281	281
Log likelihood	−279.04	−134.93	−143.60
Deviance	558.08	269.87	287.20
AIC	612.08	323.87	341.20
BIC	727.24	422.10	439.43

$^{***}p < 0.01$; $^{**}p < 0.05$; $^{*}p < 0.1$

Note: WFH=working from home; HW=housework; CC=childcare; ST=short-time

Table S6. Additional control variable: respondent is male

	Dependent variable:		
	more HW: ♂ (1)	more HW: ♂ (2)	more CC: ♂ (3)
WFH: both	0.16 (0.07)**	0.20 (0.09)**	0.12 (0.10)
WFH: only ♀	0.14 (0.10)	0.11 (0.14)	−0.03 (0.12)
WFH: only ♂	0.18 (0.11)	0.37 (0.14)***	0.29 (0.13)**
WFH: nobody (= *ref*)			
HW before: ♀ more	0.18 (0.05)***	0.15 (0.06)**	
HW before: ♀ much more	0.34 (0.06)***	0.34 (0.08)***	
HW before: ♂ (much) more	0.05 (0.07)	0.11 (0.10)	
HW before: equal (= *ref*)			
CC before: ♀ more			0.21 (0.06)***
CC before: ♀ much more			0.36 (0.07)***
CC before: ♂ (much) more			0.02 (0.12)
CC before: equal (= *ref*)			
Higher income: ♀	0.18 (0.07)***	0.25 (0.10)**	0.00 (0.11)
Higher income: ♂	0.10 (0.04)**	0.14 (0.05)**	0.06 (0.06)
Equal income (= *ref*)			
Working hours ⩽20h: ♀	−0.01 (0.05)	−0.04 (0.06)	−0.12 (0.05)**
Working hours ⩽20h (ST): ♀	−0.02 (0.07)	0.02 (0.09)	−0.03 (0.09)
Working hours >20h (ST): ♀	−0.14 (0.14)	−0.20 (0.10)**	0.05 (0.22)
Working hours >20h: ♀ (= *ref*)			
Working hours ⩽20h: ♂	0.15 (0.10)	0.38 (0.12)***	0.47 (0.09)***
Working hours ⩽20h (ST): ♂	0.05 (0.09)	0.05 (0.10)	0.15 (0.10)
Working hours >20h (ST): ♂	0.03 (0.11)	−0.20 (0.09)**	−0.25 (0.10)**
Working hours >20h: ♂ (= *ref*)			
Self-employed: ♀	−0.07 (0.06)	−0.07 (0.08)	−0.18 (0.08)**
Employed: ♀ (= *ref*)			
Self-employed: ♂	−0.06 (0.05)	−0.02 (0.07)	−0.17 (0.07)**
Employed: ♂ (= *ref*)			
No. children 0 – 2 years	−0.05 (0.05)	0.01 (0.07)	0.04 (0.07)
No. children 3 – 5 years	−0.05 (0.04)	−0.00 (0.05)	0.05 (0.06)
No. children 6 – 9 years	0.07 (0.03)**	0.10 (0.04)**	0.00 (0.05)
No. children 10 – 14 years	−0.06 (0.04)	−0.09 (0.06)	−0.17 (0.07)***
Age: ♀	−0.01 (0.00)	0.01 (0.01)	0.02 (0.01)**
Age: ♂	0.00 (0.00)	0.00 (0.01)	0.00 (0.01)
Educ. ♀ : Higher sec.	0.02 (0.05)	−0.02 (0.07)	0.07 (0.08)
Educ. ♀ : Lower sec. | prim.	−0.06 (0.06)	−0.02 (0.10)	0.15 (0.11)
Educ. ♀ : Tertiary (= *ref*)			
Educ. ♂ : Higher sec.	0.04 (0.05)	0.03 (0.06)	0.08 (0.07)
Educ. ♂ : Lower sec. | prim.	0.01 (0.06)	0.00 (0.09)	−0.08 (0.08)
Educ. ♂ : Tertiary (= *ref*)			
Info by man	0.18 (0.05)***	0.28 (0.07)***	0.12 (0.07)
Info by woman (= *ref*)			
Observations	558	299	299
Log likelihood	−293.65	−140.88	−151.61
Deviance	587.31	281.76	303.22
AIC	643.31	337.76	359.22
BIC	764.39	441.37	462.83

$^{***}p < 0.01$; $^{**}p < 0.05$; $^{*}p < 0.1$

Note: WFH=working from home; HW=housework; CC=childcare; ST=short-time

Table S7. Definition of the income variable: subjective assessment

	Dependent variable:		
	more HW: ♂ (1)	more HW: ♂ (2)	more CC: ♂ (3)
WFH: both	0.14 (0.07)*	0.19 (0.10)*	0.09 (0.11)
WFH: only ♀	0.11 (0.10)	0.09 (0.15)	−0.06 (0.12)
WFH: only ♂	0.20 (0.12)*	0.41 (0.14)***	0.31 (0.13)**
WFH: nobody (= *ref*)			
HW before: ♀ more	0.16 (0.05)***	0.15 (0.06)**	
HW before: ♀ much more	0.33 (0.06)***	0.35 (0.08)***	
HW before: ♂ (much) more	0.07 (0.07)	0.13 (0.10)	
HW before: equal (= *ref*)			
CC before: ♀ more			0.22 (0.06)***
CC before: ♀ much more			0.37 (0.07)***
CC before: ♂ (much) more			0.04 (0.13)
CC before: equal (= *ref*)			
Higher income: ♀	0.15 (0.07)**	0.15 (0.10)	0.04 (0.10)
Higher income: ♂	0.16 (0.06)***	0.21 (0.08)***	0.04 (0.09)
Equal income (= *ref*)			
Working hours ⩽20h: ♀	0.01 (0.05)	−0.01 (0.06)	−0.08 (0.06)
Working hours ⩽20h (ST): ♀	−0.02 (0.08)	−0.00 (0.09)	−0.01 (0.10)
Working hours >20h (ST): ♀	−0.13 (0.15)	−0.19 (0.11)*	0.06 (0.23)
Working hours >20h: ♀ (= *ref*)			
Working hours ⩽20h: ♂	0.20 (0.10)**	0.39 (0.12)***	0.45 (0.10)***
Working hours ⩽20h (ST): ♂	0.08 (0.09)	0.06 (0.11)	0.13 (0.11)
Working hours >20h (ST): ♂	0.01 (0.11)	−0.21 (0.08)***	−0.25 (0.10)**
Working hours >20h: ♂ (= *ref*)			
Self-employed: ♀	−0.05 (0.07)	−0.07 (0.08)	−0.16 (0.08)*
Employed: ♀ (= *ref*)			
Self-employed: ♂	−0.07 (0.05)	−0.03 (0.07)	−0.19 (0.07)***
Employed: ♂ (= *ref*)			
No. children 0 – 2 years	−0.04 (0.05)	0.01 (0.08)	0.05 (0.08)
No. children 3 – 5 years	−0.07 (0.04)	−0.04 (0.06)	0.04 (0.06)
No. children 6 – 9 years	0.05 (0.04)	0.08 (0.05)*	−0.01 (0.05)
No. children 10 – 14 years	−0.06 (0.04)	−0.08 (0.06)	−0.18 (0.07)***
Age: ♀	−0.01 (0.00)	0.00 (0.01)	0.01 (0.01)*
Age: ♂	0.00 (0.00)	0.00 (0.01)	0.00 (0.01)
Educ. ♀ : Higher sec.	0.01 (0.05)	−0.05 (0.07)	0.09 (0.08)
Educ. ♀ : Lower sec. | prim.	−0.03 (0.07)	−0.01 (0.10)	0.16 (0.11)
Educ. ♀ : Tertiary (= *ref*)			
Educ. ♂ : Higher sec.	0.03 (0.05)	0.02 (0.07)	0.07 (0.07)
Educ. ♂ : Lower sec. | prim.	−0.01 (0.06)	0.01 (0.09)	−0.10 (0.08)
Educ. ♂ : Tertiary (= *ref*)			
Observations	551	293	293
Log likelihood	−297.89	−146.75	−151.05
Deviance	595.79	293.49	302.09
AIC	649.79	347.49	356.09
BIC	766.21	446.86	455.46

$^{***}p < 0.01$; $^{**}p < 0.05$; $^{*}p < 0.1$

Note: WFH=working from home; HW=housework; CC=childcare; ST=short-time

The sample is larger due to a smaller number of missing values in the alternative income variable.

Table S8. Definition of working hours: continuous working hours

	Dependent variable:		
	more HW: ♂ (1)	more HW: ♂ (2)	more CC: ♂ (3)
WFH: both	0.1441 (0.0714)**	0.1530 (0.1000)	0.0415 (0.1069)
WFH: only ♀	0.0877 (0.0954)	0.0030 (0.1350)	−0.1374 (0.1049)
WFH: only ♂	0.2036 (0.1150)*	0.3108 (0.1525)**	0.1491 (0.1293)
WFH: nobody (= *ref*)			
HW before: ♀ more	0.1655 (0.0474)***	0.1404 (0.0638)**	
HW before: ♀ much more	0.3236 (0.0631)***	0.3268 (0.0844)***	
HW before: ♂ (much) more	0.0654 (0.0673)	0.1213 (0.1042)	
HW before: equal (= *ref*)			
CC before: ♀ more			0.2418 (0.0607)***
CC before: ♀ much more			0.4096 (0.0619)***
CC before: ♂ (much) more			0.0505 (0.1170)
CC before: equal (= *ref*)			
Higher income: ♀	0.1551 (0.0696)**	0.2202 (0.1079)**	0.0057 (0.0990)
Higher income: ♂	0.1029 (0.0444)**	0.1239 (0.0578)**	0.0960 (0.0600)
Equal income (= *ref*)			
Working hours: ♀	0.0000 (0.0022)	−0.0000 (0.0031)	0.0060 (0.0033)*
Working hours: ♂	−0.0052 (0.0020)***	−0.0057 (0.0024)**	−0.0126 (0.0032)***
Self-employed: ♀	−0.0660 (0.0607)	−0.0911 (0.0753)	−0.2036 (0.0693)***
Employed: ♀ (= *ref*)			
Self-employed: ♂	−0.0493 (0.0540)	0.0358 (0.0750)	−0.0568 (0.0772)
Employed: ♂ (= *ref*)			
No. children 0 – 2 years	−0.0532 (0.0544)	0.0228 (0.0742)	0.0370 (0.0713)
No. children 3 – 5 years	−0.0701 (0.0428)	−0.0322 (0.0550)	0.0346 (0.0549)
No. children 6 – 9 years	0.0513 (0.0341)	0.0717 (0.0436)*	−0.0102 (0.0448)
No. children 10 – 14 years	−0.0623 (0.0414)	−0.0813 (0.0576)	−0.1532 (0.0633)**
Age: ♀	−0.0049 (0.0044)	0.0050 (0.0070)	0.0143 (0.0077)*
Age: ♂	0.0036 (0.0041)	0.0032 (0.0055)	0.0020 (0.0059)
Educ. ♀ : Higher sec.	0.0222 (0.0553)	0.0139 (0.0799)	0.0969 (0.0839)
Educ. ♀ : Lower sec. | prim.	−0.0424 (0.0667)	0.0065 (0.1041)	0.1496 (0.1045)
Educ. ♀ : Tertiary (= *ref*)			
Educ. ♂ : Higher sec.	0.0109 (0.0467)	−0.0041 (0.0618)	0.0539 (0.0636)
Educ. ♂ : Lower sec. | prim.	−0.0074 (0.0557)	−0.0327 (0.0846)	−0.1096 (0.0812)
Educ. ♂ : Tertiary (= *ref*)			
Observations	558	299	299
Log likelihood	−297.3819	−149.3647	−149.2766
Deviance	594.7638	298.7295	298.5531
AIC	640.7638	344.7295	344.5531
BIC	740.2241	429.8397	429.6633

$^{***}p < 0.01$; $^{**}p < 0.05$; $^{*}p < 0.1$

Note: WFH=working from home; HW=housework; CC=childcare; ST=short-time

Table S9. Definition of working hours: part-time $\leqslant 35h$

	Dependent variable:		
	more HW: ♂ (1)	more HW: ♂ (2)	more CC: ♂ (3)
WFH: both	0.15 (0.07)**	0.18 (0.10)*	0.10 (0.10)
WFH: only ♀	0.10 (0.10)	0.03 (0.14)	−0.06 (0.12)
WFH: only ♂	0.22 (0.12)*	0.36 (0.15)**	0.24 (0.13)*
WFH: nobody (= *ref*)			
HW before: ♀ more	0.16 (0.05)***	0.15 (0.06)**	
HW before: ♀ much more	0.32 (0.06)***	0.35 (0.08)***	
HW before: ♂ (much) more	0.06 (0.07)	0.12 (0.10)	
HW before: equal (= *ref*)			
CC before: ♀ more			0.23 (0.06)***
CC before: ♀ much more			0.39 (0.07)***
CC before: ♂ (much) more			0.05 (0.12)
CC before: equal (= *ref*)			
Higher income: ♀	0.16 (0.07)**	0.22 (0.11)**	0.03 (0.11)
Higher income: ♂	0.09 (0.04)**	0.12 (0.06)**	0.05 (0.06)
Equal income (= *ref*)			
Working hours ⩽35h: ♀	0.01 (0.05)	0.00 (0.07)	−0.01 (0.08)
Working hours ⩽35h (ST): ♀	−0.03 (0.08)	−0.05 (0.10)	−0.02 (0.11)
Working hours >35h: ♀ (= *ref*)			
Working hours ⩽35h: ♂	0.08 (0.05)	0.16 (0.07)**	0.21 (0.07)***
Working hours ⩽35h (ST): ♂	0.08 (0.08)	0.02 (0.10)	0.11 (0.10)
Working hours >35h: ♂ (= *ref*)			
Self-employed: ♀	−0.06 (0.06)	−0.10 (0.07)	−0.21 (0.07)***
Employed: ♀ (= *ref*)			
Self-employed: ♂	−0.07 (0.05)	−0.01 (0.07)	−0.12 (0.07)
Employed: ♂ (= *ref*)			
No. children 0 – 2 years	−0.05 (0.05)	0.02 (0.07)	0.04 (0.07)
No. children 3 – 5 years	−0.07 (0.04)*	−0.05 (0.06)	0.01 (0.06)
No. children 6 – 9 years	0.05 (0.03)	0.07 (0.04)*	−0.02 (0.05)
No. children 10 – 14 years	−0.06 (0.04)	−0.08 (0.06)	−0.17 (0.07)**
Age: ♀	−0.00 (0.00)	0.01 (0.01)	0.02 (0.01)**
Age: ♂	0.00 (0.00)	0.00 (0.01)	0.00 (0.01)
Educ. ♀ : Higher sec.	0.03 (0.06)	0.01 (0.08)	0.11 (0.09)
Educ. ♀ : Lower sec. | prim.	−0.04 (0.07)	0.02 (0.11)	0.15 (0.11)
Educ. ♀ : Tertiary (= *ref*)			
Educ. ♂ : Higher sec.	0.02 (0.05)	0.01 (0.06)	0.07 (0.07)
Educ. ♂ : Lower sec. | prim.	0.00 (0.06)	−0.00 (0.09)	−0.07 (0.09)
Educ. ♂ : Tertiary (= *ref*)			
Observations	558	299	299
Log likelihood	−299.47	−149.76	−158.13
Deviance	598.93	299.53	316.26
AIC	648.93	349.53	366.26
BIC	757.04	442.04	458.77

$^{***}p < 0.01$; $^{**}p < 0.05$; $^{*}p < 0.1$

Note: WFH=working from home; HW=housework; CC=childcare; ST=short-time

Table S10. Definition of age variable: age groups

	Dependent variable:		
	more HW: ♂ (1)	more HW: ♂ (2)	more CC: ♂ (3)
WFH: both	0.15 (0.07)**	0.19 (0.10)**	0.14 (0.10)
WFH: only ♀	0.10 (0.10)	0.04 (0.15)	−0.03 (0.12)
WFH: only ♂	0.22 (0.12)*	0.42 (0.13)***	0.35 (0.12)***
WFH: nobody (= *ref*)			
HW before: ♀ more	0.17 (0.05)***	0.15 (0.06)**	
HW before: ♀ much more	0.33 (0.06)***	0.33 (0.08)***	
HW before: ♂ (much) more	0.07 (0.07)	0.17 (0.11)	
HW before: equal (= *ref*)			
CC before: ♀ more			0.20 (0.07)***
CC before: ♀ much more			0.35 (0.07)***
CC before: ♂ (much) more			0.05 (0.12)
CC before: equal (= *ref*)			
Higher income: ♀	0.18 (0.07)**	0.23 (0.11)**	−0.00 (0.11)
Higher income: ♂	0.09 (0.04)**	0.11 (0.06)*	0.06 (0.06)
Equal income (= *ref*)			
Working hours ⩽20h: ♀	0.01 (0.05)	−0.03 (0.06)	−0.13 (0.06)**
Working hours ⩽20h (ST): ♀	−0.02 (0.08)	0.01 (0.10)	−0.07 (0.09)
Working hours >20h (ST): ♀	−0.14 (0.15)	−0.18 (0.11)	0.05 (0.21)
Working hours >20h: ♀ (= *ref*)			
Working hours ⩽20h: ♂	0.16 (0.10)	0.35 (0.13)***	0.49 (0.09)***
Working hours ⩽20h (ST): ♂	0.07 (0.09)	0.03 (0.10)	0.16 (0.11)
Working hours >20h (ST): ♂	0.01 (0.10)	−0.18 (0.10)*	−0.23 (0.11)**
Working hours >20h: ♂ (= *ref*)			
Self-employed: ♀	−0.05 (0.06)	−0.10 (0.07)	−0.19 (0.08)**
Employed: ♀ (= *ref*)			
Self-employed: ♂	−0.08 (0.05)	−0.03 (0.07)	−0.16 (0.07)**
Employed: ♂ (= *ref*)			
No. children 0 – 2 years	−0.05 (0.06)	0.01 (0.08)	0.02 (0.07)
No. children 3 – 5 years	−0.07 (0.04)	−0.02 (0.05)	0.04 (0.06)
No. children 6 – 9 years	0.05 (0.04)	0.10 (0.05)**	−0.01 (0.05)
No. children 10 – 14 years	−0.07 (0.04)	−0.07 (0.06)	−0.15 (0.06)**
Age group 18 – 29: ♀	0.06 (0.08)	0.03 (0.15)	−0.08 (0.12)
Age group 40 – 49: ♀	−0.01 (0.06)	0.01 (0.07)	0.06 (0.07)
Age group 50 – 59: ♀	−0.08 (0.07)	0.17 (0.15)	0.25 (0.14)*
Age group >59: ♀	−0.19 (0.10)*		
Age group 30 – 39: ♀ (= *ref*)			
Age group 18 – 29: ♂	−0.06 (0.08)	−0.19 (0.10)*	−0.19 (0.13)
Age group 40 – 49: ♂	0.01 (0.06)	−0.03 (0.07)	0.09 (0.07)
Age group 50 – 59: ♂	0.05 (0.08)	−0.04 (0.09)	−0.06 (0.10)
Age group >59: ♂	0.04 (0.13)	0.23 (0.37)	0.21 (0.32)
Age group 30 – 39: ♂ (= *ref*)			
Educ. ♀ : Higher sec.	0.03 (0.06)	0.01 (0.08)	0.12 (0.09)
Educ. ♀ : Lower sec. | prim.	−0.04 (0.07)	0.02 (0.11)	0.23 (0.11)**
Educ. ♀ : Tertiary (= *ref*)			
Educ. ♂ : Higher sec.	0.02 (0.05)	0.01 (0.06)	0.08 (0.07)
Educ. ♂ : Lower sec. | prim.	−0.01 (0.06)	−0.04 (0.08)	−0.12 (0.08)
Educ. ♂ : Tertiary (= *ref*)			
Observations	558	299	299
Log likelihood	−298.45	−147.20	−152.44
Deviance	596.89	294.40	304.87
AIC	662.89	358.40	368.87
BIC	805.60	476.81	487.29

$^{***}p < 0.01$; $^{**}p < 0.05$; $^{*}p < 0.1$

Note: WFH=working from home; HW=housework; CC=childcare; ST=short-time

Table S11. Definition of children living in the household: age youngest child

	Dependent variable:		
	more HW: ♂ (1)	more HW: ♂ (2)	more CC: ♂ (3)
WFH: both	0.15 (0.07)**	0.19 (0.10)*	0.12 (0.10)
WFH: only ♀	0.11 (0.10)	0.06 (0.15)	−0.03 (0.12)
WFH: only ♂	0.22 (0.12)*	0.41 (0.14)***	0.32 (0.12)**
WFH: nobody (= *ref*)			
HW before: ♀ more	0.17 (0.05)***	0.15 (0.06)**	
HW before: ♀ much more	0.33 (0.06)***	0.34 (0.08)***	
HW before: ♂ (much) more	0.07 (0.07)	0.16 (0.11)	
HW before: equal (= *ref*)			
CC before: ♀ more			0.23 (0.06)***
CC before: ♀ much more			0.36 (0.07)***
CC before: ♂ (much) more			0.04 (0.12)
CC before: equal (= *ref*)			
Higher income: ♀	0.17 (0.07)**	0.19 (0.11)*	−0.01 (0.10)
Higher income: ♂	0.09 (0.04)**	0.10 (0.06)*	0.04 (0.06)
Equal income (= *ref*)			
Working hours ⩽20h: ♀	0.01 (0.05)	−0.01 (0.06)	−0.11 (0.06)*
Working hours ⩽20h (ST): ♀	−0.03 (0.07)	−0.02 (0.09)	−0.06 (0.09)
Working hours >20h (ST): ♀	−0.11 (0.16)	−0.15 (0.14)	0.06 (0.22)
Working hours >20h: ♀ (= *ref*)			
Working hours ⩽20h: ♂	0.16 (0.10)	0.34 (0.13)**	0.47 (0.09)***
Working hours ⩽20h (ST): ♂	0.08 (0.09)	0.05 (0.10)	0.14 (0.11)
Working hours >20h (ST): ♂	0.01 (0.10)	−0.20 (0.09)**	−0.28 (0.08)***
Working hours >20h: ♂ (= *ref*)			
Self-employed: ♀	−0.06 (0.06)	−0.09 (0.08)	−0.19 (0.08)**
Employed: ♀ (= *ref*)			
Self-employed: ♂	−0.07 (0.05)	−0.05 (0.07)	−0.19 (0.07)***
Employed: ♂ (= *ref*)			
Age youngest child: 3 – 5 years	0.02 (0.07)	−0.01 (0.07)	0.01 (0.07)
Age youngest child: 6 – 9 years	0.09 (0.08)	0.04 (0.08)	−0.07 (0.08)
Age youngest child: 10 – 14 years	0.02 (0.08)	−0.12 (0.09)	−0.24 (0.07)***
Age youngest child: No child < 15 years	0.08 (0.06)		
Age youngest child: 0 – 2 years (= *ref*)			
Age: ♀	−0.01 (0.00)	0.01 (0.01)	0.02 (0.01)**
Age: ♂	0.00 (0.00)	0.00 (0.01)	0.00 (0.01)
Educ. ♀ : Higher sec.	0.03 (0.06)	−0.00 (0.08)	0.09 (0.09)
Educ. ♀ : Lower sec. | prim.	−0.05 (0.07)	−0.01 (0.10)	0.17 (0.11)
Educ. ♀ : Tertiary (= *ref*)			
Educ. ♂ : Higher sec.	0.02 (0.05)	−0.00 (0.06)	0.07 (0.07)
Educ. ♂ : Lower sec. | prim.	0.00 (0.06)	−0.00 (0.09)	−0.07 (0.09)
Educ. ♂ : Tertiary (= *ref*)			
Observations	558	299	299
Log Likelihood	−301.70	−151.29	−154.90
Deviance	603.40	302.59	309.81
AIC	657.40	354.59	361.81
BIC	774.16	450.80	458.02

$^{***}p < 0.01$; $^{**}p < 0.05$; $^{*}p < 0.1$

Note: WFH=working from home; HW=housework; CC=childcare; ST=short-time

Table S12. Definition of the dependent variable: woman works more

	Dependent variable:		
	more HW: ♀ (1)	more HW: ♀ (2)	more CC: ♀ (3)
WFH: both	−0.11 (0.08)	−0.04 (0.11)	−0.03 (0.10)
WFH: only ♀	−0.00 (0.08)	0.09 (0.12)	0.12 (0.12)
WFH: only ♂	−0.04 (0.08)	0.10 (0.14)	−0.03 (0.13)
WFH: nobody (= *ref*)			
HW before: ♀ more	0.05 (0.05)	−0.01 (0.06)	
HW before: ♀ much more	−0.18 (0.05)***	−0.27 (0.06)***	
HW before: ♂ (much) more	0.18 (0.06)***	0.01 (0.09)	
HW before: equal (= *ref*)			
CC before: ♀ more			−0.03 (0.06)
CC before: ♀ much more			−0.38 (0.05)***
CC before: ♂ (much) more			0.00 (0.09)
CC before: equal (= *ref*)			
Higher income: ♀	−0.05 (0.06)	−0.10 (0.09)	−0.04 (0.10)
Higher income: ♂	0.03 (0.04)	0.01 (0.06)	0.06 (0.06)
Equal income (= *ref*)			
Working hours ⩽20h: ♀	0.02 (0.05)	0.06 (0.07)	0.06 (0.06)
Working hours ⩽20h (ST): ♀	0.17 (0.09)**	0.12 (0.11)	0.02 (0.10)
Working hours >20h (ST): ♀	0.20 (0.28)	0.29 (0.27)	0.31 (0.21)
Working hours >20h: ♀ (= *ref*)			
Working hours ⩽20h: ♂	−0.06 (0.08)	−0.18 (0.10)*	−0.22 (0.09)**
Working hours ⩽20h (ST): ♂	0.05 (0.08)	−0.05 (0.11)	−0.01 (0.10)
Working hours >20h (ST): ♂	−0.09 (0.09)	−0.05 (0.17)	−0.01 (0.17)
Working hours >20h: ♂ (= *ref*)			
Self-employed: ♀	−0.01 (0.07)	0.05 (0.10)	0.07 (0.10)
Employed: ♀ (= *ref*)			
Self-employed: ♂	0.05 (0.06)	0.05 (0.09)	−0.06 (0.08)
Employed: ♂ (= *ref*)			
No. children 0 – 2 years	0.10 (0.05)**	−0.07 (0.08)	−0.00 (0.08)
No. children 3 – 5 years	0.09 (0.04)**	−0.04 (0.06)	−0.06 (0.06)
No. children 6 – 9 years	−0.03 (0.04)	−0.11 (0.05)**	0.07 (0.05)
No. children 10 – 14 years	0.06 (0.04)*	−0.04 (0.06)	0.08 (0.06)
Age: ♀	0.01 (0.00)	0.01 (0.01)	−0.01 (0.01)
Age: ♂	−0.01 (0.00)*	−0.02 (0.01)**	−0.01 (0.01)
Educ. ♀ : Higher sec.	−0.05 (0.05)	−0.01 (0.09)	−0.01 (0.09)
Educ. ♀ : Lower sec. | prim.	−0.04 (0.07)	−0.02 (0.10)	−0.04 (0.10)
Educ. ♀ : Tertiary (= *ref*)			
Educ. ♂ : Higher sec.	0.04 (0.05)	0.06 (0.07)	−0.12 (0.06)*
Educ. ♂ : Lower sec. | prim.	−0.08 (0.05)	−0.11 (0.09)	−0.11 (0.08)
Educ. ♂ : Tertiary (= *ref*)			
Observations	558	299	299
Log likelihood	−296.06	−170.22	−160.10
Deviance	592.12	340.44	320.20
AIC	646.12	394.44	374.20
BIC	762.88	494.35	474.12

***$p < 0.01$; **$p < 0.05$; *$p < 0.1$

Note: WFH=working from home; HW=housework; CC=childcare; ST=short-time

Table S13. Definition of the dependent variable: more equal division of unpaid work within the household

	Dependent variable:		
	more equal HW (1)	more equal HW (2)	more equal CC (3)
WFH: both	0.11 (0.06)*	0.16 (0.07)**	0.22 (0.08)***
WFH: only ♀	−0.03 (0.08)	0.01 (0.12)	0.07 (0.12)
WFH: only ♂	0.14 (0.10)	0.33 (0.12)***	0.28 (0.10)***
WFH: nobody (= *ref*)			
HW before: equal	−0.34 (0.03)***	−0.30 (0.03)***	
HW before: ♀ more	−0.10 (0.04)***	−0.13 (0.05)***	
HW before: ♂ (much) more	−0.12 (0.04)***	−0.10 (0.05)*	
HW before: ♀ much more (= *ref*)			
CC before: equal			−0.39 (0.03)***
CC before: ♀ more			−0.13 (0.05)**
CC before: ♂ (much) more			−0.11 (0.07)
CC before: ♀ much more (= *ref*)			
Higher income: ♀	0.01 (0.06)	−0.06 (0.09)	0.04 (0.11)
Higher income: ♂	0.05 (0.04)	0.13 (0.05)***	0.06 (0.06)
Equal income (= *ref*)			
Working hours ⩽20h: ♀	−0.01 (0.04)	−0.05 (0.05)	−0.04 (0.06)
Working hours ⩽20h (ST): ♀	−0.09 (0.05)*	−0.09 (0.06)	−0.01 (0.09)
Working hours >20h (ST): ♀	−0.22 (0.02)***	−0.21 (0.02)***	0.10 (0.18)
Working hours >20h: ♀ (= *ref*)			
Working hours ⩽20h: ♂	0.17 (0.09)*	0.38 (0.09)***	0.26 (0.10)**
Working hours ⩽20h (ST): ♂	0.02 (0.08)	0.06 (0.10)	0.02 (0.10)
Working hours >20h (ST): ♂	0.02 (0.09)	−0.11 (0.10)	−0.17 (0.11)
Working hours >20h: ♂ (= *ref*)			
Self-employed: ♀	0.01 (0.06)	−0.06 (0.06)	−0.22 (0.06)***
Employed: ♀ (= *ref*)			
Self-employed: ♂	−0.08 (0.05)*	−0.01 (0.07)	−0.14 (0.07)**
Employed: ♂ (= *ref*)			
No. children 0 – 2 years	−0.01 (0.04)	−0.03 (0.07)	0.07 (0.07)
No. children 3 – 5 years	−0.07 (0.04)*	−0.07 (0.05)	0.03 (0.05)
No. children 6 – 9 years	−0.02 (0.03)	−0.02 (0.04)	−0.03 (0.04)
No. children 10 – 14 years	−0.06 (0.04)	−0.12 (0.06)**	−0.07 (0.05)
Age: ♀	0.00 (0.00)	0.00 (0.01)	0.01 (0.01)
Age: ♂	−0.00 (0.00)	0.00 (0.00)	0.00 (0.01)
Educ. ♀ : Higher sec.	0.01 (0.05)	0.04 (0.07)	0.06 (0.08)
Educ. ♀ : Lower sec. | prim.	−0.02 (0.06)	0.11 (0.10)	0.09 (0.10)
Educ. ♀ : Tertiary (= *ref*)			
Educ. ♂ : Higher sec.	0.04 (0.04)	0.03 (0.05)	0.01 (0.06)
Educ. ♂ : Lower sec. | prim.	0.02 (0.05)	−0.00 (0.08)	−0.00 (0.09)
Educ. ♂ : Tertiary (= *ref*)			
Observations	558	299	299
Log likelihood	−227.00	−106.88	−130.15
Deviance	454.01	213.76	260.30
AIC	508.01	267.76	314.30
BIC	624.76	367.67	414.21

$^{***}p < 0.01$; $^{**}p < 0.05$; $^{*}p < 0.1$

Note: WFH=working from home; HW=housework; CC=childcare; ST=short-time

Table S14. Linear probability model

	Dependent variable:		
	more HW: ♀	more HW: ♀	more CC: ♀
	(1)	(2)	(3)
WFH: both	0.15 (0.07)**	0.19 (0.10)**	0.11 (0.10)
WFH: only ♀	0.11 (0.10)	0.06 (0.15)	−0.04 (0.12)
WFH: only ♂	0.23 (0.12)**	0.42 (0.14)***	0.30 (0.12)**
WFH: nobody (= *ref*)			
HW before: ♀ more	0.17 (0.05)***	0.15 (0.06)**	
HW before: ♀ much more	0.32 (0.06)***	0.33 (0.08)***	
HW before: ♂ (much) more	0.07 (0.07)	0.14 (0.11)	
HW before: ♀ much more (= *ref*)			
CC before: ♀ more			0.21 (0.06)***
CC before: ♀ much more			0.36 (0.07)***
CC before: ♂ (much) more			0.04 (0.12)
CC before: ♀ much more (= *ref*)			
Higher income: ♀	0.17 (0.07)**	0.21 (0.11)*	−0.01 (0.10)
Higher income: ♂	0.09 (0.04)**	0.11 (0.06)**	0.06 (0.06)
Equal income (= *ref*)			
Working hours <=20h: ♀	0.01 (0.05)	−0.01 (0.06)	−0.11 (0.06)*
Working hours <=20h (ST): ♀	−0.02 (0.08)	0.00 (0.09)	−0.03 (0.09)
Working hours >20h (ST): ♀	−0.14 (0.15)	−0.18 (0.11)*	0.05 (0.22)
Working hours >20h: ♀ (= *ref*)			
Working hours <=20h: ♂	0.15 (0.10)	0.34 (0.13)***	0.46 (0.09)***
Working hours <=20h (ST): ♂	0.06 (0.09)	0.03 (0.10)	0.14 (0.11)
Working hours >20h (ST): ♂	0.01 (0.10)	−0.19 (0.09)**	−0.25 (0.10)**
Working hours >20h: ♂ (= *ref*)			
Self-employed: ♀	−0.06 (0.06)	−0.09 (0.08)	−0.20 (0.07)***
Employed: ♀ (= *ref*)			
Self-employed: ♂	−0.07 (0.05)	−0.04 (0.07)	−0.18 (0.07)***
Employed: ♂ (= *ref*)			
No. children 0 – 2 years	−0.05 (0.05)	0.03 (0.07)	0.05 (0.07)
No. children 3 – 5 years	−0.06 (0.04)	−0.02 (0.05)	0.05 (0.06)
No. children 6 – 9 years	0.06 (0.03)*	0.09 (0.04)*	0.00 (0.05)
No. children 10 – 14 years	−0.06 (0.04)	−0.09 (0.06)	−0.17 (0.07)**
Age: ♀	−0.00 (0.00)	0.01 (0.01)	0.02 (0.01)**
Age: ♂	0.00 (0.00)	0.00 (0.01)	0.00 (0.01)
Educ. ♀ : Higher sec.	0.02 (0.06)	−0.00 (0.08)	0.09 (0.08)
Educ. ♀ : Lower sec. | prim.	−0.04 (0.07)	0.01 (0.10)	0.16 (0.11)
Educ. ♀ : Tertiary (= *ref*)			
Educ. ♂ : Higher sec.	0.02 (0.05)	0.00 (0.06)	0.06 (0.06)
Educ. ♂ : Lower sec. | prim.	−0.00 (0.06)	−0.02 (0.09)	−0.09 (0.08)
Educ. ♂ : Tertiary (= *ref*)			
Observations	558	299	299
Log likelihood	−299.55	−148.15	−152.85
Deviance	599.09	296.30	305.70
AIC	653.09	350.30	359.70
BIC	769.85	450.21	459.61

$^{***}p < 0.01$; $^{**}p < 0.05$; $^{*}p < 0.1$

Note: WFH=working from home; HW=housework; CC=childcare; ST=short-time
