# Supplementary material for: Home, sweet home? The impact of working from home on the division of unpaid work during the COVID-19 lockdown
Source: PLoS One. 2021 Nov 8;16(11):e0259580. doi: 10.1371/journal.pone.0259580 (PMC8575241; doi:10.1371/journal.pone.0259580)
Supplement: Attachment — Submitted filename: responses.pdf [file pone.0259580.s002.pdf]

Dear Niaz Asadullah,
Dear Reviewer,

Thank you for giving us the opportunity to submit a revised version of our manuscript titled *'Home, sweet home? The impact of working from home on the division of unpaid work during the COVID-19 lockdown'* to PLoS ONE to be considered for publication as *Research Article.*

We appreciate the effort and time the editor and the reviewer have dedicated in providing valuable feedback on the manuscript and we are grateful for all comments. We have incorporated adaptions that reflect upon the suggestions and we have been able to address all the issues raised in the feedback. The remarks have been immensely helpful and we are convinced that the manuscript improved substantially.

We include and discuss the comments provided by the reviewer and the editor immediately on the follow-up to this letter. We reply to them individually, indicating exactly how we addressed each concern or problem, and the responses describe all the incorporated changes we have made. The revisions have been approved by all authors. In the *marked* copy of the manuscript and supporting material, additions to the text are highlighted in red, while words, sentences or paragraphs we have excluded are ~~crossed out~~. We also submitted an *unmarked* and standardized version of the revised manuscript and supporting material. We also respond to the additional journal requirements in this letter.

Please address all correspondence concerning this manuscript to me at franziska.disslbacher@wu.ac.at.

We are very much looking forward to your decision and greatly appreciate your attention and valuable time.

Yours sincerely,

Franziska Disslbacher

Co-Authors: Judith Derndorfer, Vanessa Lechinger, Katharina Mader and Eva Six

Comments provided by the reviewer (RC)

RC1: The authors could provide a better sense of how much more unpaid work women do (globally and specifically to Austria/similar countries) rather than just stating women do more unpaid work.

Response to RC1: We agree that this is important information for the reader. We have included a discussion of how much more unpaid work women do globally and in Austria, as compared to similar high-income countries in section 1 "Introduction" of the manuscript. The paragraph reads as follows:

"Based on a collection of 133 time use surveys carried out in 76 countries during the past 20 years, Charmes (2019) reports that globally "women carry out three-quarters of unpaid care work, or more than 75% of the total hours provided" (ibid., p.3). This most comprehensive study, in terms of the world's population covered, concludes that there is not a single country where women and men perform an equal share of unpaid care work. Across countries, women's share in unpaid work ranges from 55.3% in Sweden to 92% in Mali. It is only in three Scandinavian countries (Sweden, Norway and Denmark), where the female share of unpaid work is below 60%. According to that report, women conducted 64.8% of unpaid work in Austria in 2008/2009 which is a substantially higher fraction as compared to Scandinavia and other high-income countries, such as Canada. From a global perspective, Austria is located among the half of countries where the women's share of unpaid work relative to their total work share, paid and unpaid, is particularly high (62.7% of total work). These numbers are roughly in line with a report of Statistics Austria that is based on the same data, namely the latest time use survey conducted in Austria in 2008/2009 (Statistics Austria, 2016). It finds that women in Austria do two thirds of unpaid work and one third of paid work."

RC2: Firstly I would like to see much more discussion of the gender bargaining literature, how this paper fits in and a clear theoretical framework.

Response to RC2: Thank you for this suggestion. We are convinced that such a discussion will help to better connect the manuscript to the larger debate on bargaining within households, and to studies on gender(-ed) norms as determinants of gender gaps in paid and unpaid work. Please note that we do not observe the bargaining process itself, but the outcome of the process. We consider this to be an important caveat in relation to the gender bargaining literature. However, we included a discussion of bargaining models and the empirical studies testing the predictions of these models. Based on theoretical considerations, we argue that the division of unpaid work within households results from established practices and gender specific social norms. The corresponding paragraph in section 1 "Introduction" of the manuscript reads as follows:

"In the economic literature, the notion of asymmetric intra-household bargaining as a mechanism that causes an unequal distribution of both paid and unpaid work among household members appeared first in the bargaining models of Manser and Brown (1980) and McElroy and Horney (1981). Bargaining models describe the intra-household allocation of resources as an outcome of a rational bargaining processes, and the individual household members are recognized as separate agents, each having their own preferences and a distinct utility function. The many models that have been suggested differ in their assumptions on the determinants of individual bargaining power, but typically, the access to economic resources, such as earnings or wealth, is emphasized as a critical determinant of an individual's degree of power (see references [19, 27-29] in the manuscript). Evidence on the resources subject to bargaining and the consequences of intra-household decision making exists primarily for countries located in the Global South (see references [30,31] in the manuscript), for instance, in terms of decisions over health and nutrition (see references [32-34] in the manuscript). For European countries, there is less empirical evidence on intra-household dynamics and the distribution of decision-making power within households. However, Dema-Moreno (2009) studies the decision-making processes of Spanish couples, while Lyngstad et al. (2011) focus on Norwegian couples, Mader et al. (2012) investigate the gendered distribution of income and decision-making power in Austrian households, and Sikorski and Kuchler (2012) study decision-making in German households. Note that an important caveat of the present study is that we investigate and observe the outcome of bargaining and decision making processes, in contrast to studies on the process itself (for example Mader et al. 2012). The aforementioned empirical studies reveal that decisions on the allocation of time and money within the household are frequently made spontaneously, result from established practice, or have an outcome that conforms to social norms while support for actual and rational bargaining is limited. Overall, these findings suggest that women and men take on different tasks based on prevailing gender roles and gendered attributions, and that social norms encourage women to assume the primary care-taking role and to conduct the bulk of unpaid housework."

RC3: I feel it was admirable to collect time use data but I felt these were under-utilised.

Response to RC3: Thank you for pointing this out. The manuscript and the supporting document now include an extended discussion of time use by gender during the lockdown. The discussion in the manuscript is located at the beginning of section 3.1.2 "Division of unpaid work", and should result in a better insight in the amount of time spent on different activities during the lockdown. It highlights the differences in time use by gender. The corresponding paragraph in this section now reads as follows:

"However, we asked respondents how they and their partner spent the previous working day during the lockdown (see Table S3). The results reveal that women in working couples spend, on average, almost two hours more on unpaid work than men (4h03 compared to 5h58) per working day. The average time spent on unpaid work by women amounts to 6h44, compared to 7h48 for men. Table S3 in the supporting material also includes and discusses information on differences in the participation rates (i.e. the share of respondents having spent some time on a certain activity) between men and women during the lockdown. Overall, we conclude that unusual times do not translate into unusual time use by gender."

The corresponding table and the more extensive description can be found in the supporting material document (see Table S3: "Average time spent per activity during the lockdown by gender"). For the sake of completeness we included this table at the end of this letter (see Table 2) and the corresponding description of the supporting material now reads as follows:

"We asked respondents how they and their partner spent the previous working day during the lockdown, summarized in Table 2. The results reveal that women in working couples spent, on average, almost two hours more on unpaid work than men (4h03 compared to 5h58) per day. The average time spent on paid work by women amounts to 6h44 compared to 7h48 for men. It is revealing to look beyond the average time spent on a specific activity by carefully examining differences in the participation rates (i.e. the share of respondents having spent some time on a certain activity) and the average time spent on distinct activities based on this "participating" sub sample. We find that 75% of men have participated in housework activities, such as cooking and cleaning on their previous working day, whereas almost all women have done some housework. Comparing men and women who have participated in housework activities, we observe that these women spent around 25 minutes more on these tasks. For childcare activities, we also note large gender differences. Half of the woman participated in physical childcare (feeding, washing and supervision), and these women spent on average 1h50 on this activity. Among men, both the participation rate (44%) and the average time spent on taking care of children (1h29) was slightly lower. The gender difference is much larger for home-schooling related activities. More than one third of of mothers studied with their children, compared to one forth of fathers, and they spent roughly half an hour more on home-schooling than fathers (1h44 compared to 1h15). The mean time spent on childcare, among those having done any childcare activity, amounts to 3h17 for fathers and 4h43 for mothers. Looking at the time spent on childcare, housework and paid work during the lockdown jointly reveals very long overall working days for parents, in particular for mothers."

RC4: I completely understand the reasons behind not being able to see the change in hours but I find this a big limitation - is there any way to combine the time use data and who does more to get sense of how much the men who do more are really doing? Men could double their unpaid work but if this is from 1 hour to 2 hours this is not much of a change...

Response to RC4: Thank you for stressing this point. We acknowledge throughout the paper that we did not survey time use before lockdown measured in hours/minutes. We refrained from doing so for several reasons that we mention in a footnote in the paper. This note (no. 6) appears in the section "Notes" at the end of the manuscript and reads as follows:

"We refrained from surveying time use before the lockdown for the following reasons: first, several studies show that the respondents' memory of past events decreases with the time gap between the reference period and the timing of the interview, that is, recall bias increases. Reliable answers on pre-lockdown time use are unlikely, as their last working day was at least four to five weeks before the survey was released. Second, as filling out a time use model on a working day before the lockdown involves the provision of mental capacities and time of the respondents, we expected that the share of attrition, that is, the share of respondents not filling out the entire questionnaire, would be much higher in that case. In addition, it might have decreased the accuracy of

answers to questions following the time use module substantially. Hence, the gains of the module might not outweigh the effort costs of the respondents finishing the survey properly."

Instead, respondents had to report if their partner did more housework or more childcare during the lockdown compared to the division before the lockdown (see response to RC6). The four corresponding questions were:

- "Please describe the distribution of housework (cooking, cleaning, household repairs, gardening, etc.) between you and your partner (i) before and (ii) during the lockdown."
- "Please describe the distribution of childcare between you and your partner (iii) before and (iv) during the lockdown."

We are convinced that surveying the pre-lockdown distribution of housework and childcare is less prone to measurement error as compared to a question that requires respondents to recall the exact hours (or minutes) worked on a typically working day (at least) four weeks earlier. We argue that consciously thinking about categorical changes in the division of unpaid work is a more informative measure than to compare changes in minutes/hours.

RC5: Do we know how many of the respondents worked from home before the lockdown or had the option to?

Response to RC5: This is an important remark. Unfortunately, we have not surveyed whether respondents were able to WFH before the lockdown. However, we were able to include some general information on the prevalence of WFH in Austria before the outspread of the COVID-19 pandemic. This information is based on a module from the Austrian Labour Force Survey 2019 (LFS), which provides the most recent and most comprehensive data on WFH (before the lockdown). This LFS 2019 module on work organization and working time arrangements shows that the *home* as the main place of work was very rare prior to the lockdown in Austria, in particular among employees. We can not rule out the possibility, that some of the self-employed respondents were already WFH prior to the lockdown for most of the week, but we capture this by controlling for the employment status in the regressions. To sum up, we have added the following sentences in section 2.1 "The first lockdown" of the manuscript:

"Prior to the lockdown working primarily from home was not widespread in Austria: before the outbreak of the pandemic, merely 2.6% of workers have been working primarily from home. However, there is substantial variation by employment status. While WFH was more common among self-employed (13.1%), it was a very rare practice among employees (1.1%) (Statistics Austria, 2019)."

RC6: I'm not sure I fully understand how pre-lockdown division is captured.

Response to RC6: Thank you very much for pointing this out. We reformulated the explanation of how we capture the pre-lockdown division of unpaid work within households in section 2.3 "Sample and key variables" and section 3.1 "Descriptive results". We hope that it is clear now in the mentioned chapters.
In general, the measure for the division of unpaid work before and during the lockdown is based on a question that asked respondents to rank, separately, their share in total housework (HW) and childcare (CC) on a scale ranging from zero ("I do everything") to ten ("My partner does everything") in steps of one. The middle of the scale (no. 5) indicates an equal division of tasks between partners. The corresponding questions were:

- "Please describe the distribution of housework (cooking, cleaning, household repairs, gardening, etc.) between you and your partner before and during the lockdown."
- "Please describe the distribution of childcare between you and your partner before and during the lockdown."

Subsequently, we re-coded the answers given to these questions using information on the respondent's gender. This results in four variables, each measured on an 11-point-scales that ranges from zero ("Woman does everything") to ten ("Man does everything"), for housework before (HW.i) and during the lockdown (HW.ii), and childcare before (CC.i) and during the lockdown (CC.ii). (For further information please see Table S2 in the supporting material)

We use the measures of the division of unpaid work for gaining several insights. First, we use this information for the descriptive analysis (see Figure 3 in the manuscript and Figure S3 in the supporting material) to show the division of housework and childcare before COVID-19 and subsequent changes during the lockdown. Second,

we use the information of these questions for the econometric analysis to describe the pre-lockdown division of unpaid work and to generate two distinct dependent variables. The dependent variables in the econometric analysis are two dummy variables indicating whether the male partner took on more housework (dependent variable one) or more childcare tasks (dependent variable two) during the lockdown than before. These dummies are equal to one if the value on the corresponding 11-point-scale is at least one point higher during the lockdown than before. To capture the pre-lockdown division of housework and childcare in our regression model, we subdivided the 11-point-scale for housework and childcare before the lockdown (HW.i and CC.i) into four categories, i.e. four brackets: (a)"Woman does much more" (scale nos. 0–2), (b)"Woman does more" (scale nos. 3–4), (c)"Equal" (scale no. 5), (d)"Man does (much) more" (scale nos. 6–10).

We added reading examples and a more detailed explanation of the underlying data to our paper (see also RC 8). The corresponding paragraph named "Division of unpaid work within the couple household before and during the lockdown" in section 2.3 "Sample and key variables" in the manuscript now reads as follows:

"We measure the division of housework (HW) and childcare tasks (CC) before (HW.i and CC.i) and during (HW.ii and CC.ii) the lockdown separately. Respondents hence had to answer four distinct questions and rank their share of housework (HW.i and HW.ii) and childcare tasks (CC.i and CC.ii) on a scale from zero (indicating the "Woman does everything") to ten (indicating the "Man does everything") (see Table S2). In the middle (scale no. 5), the division of tasks is shared equally between partners. Housework includes cooking, shopping and cleaning, but also tasks like gardening, animal care or repair work. Childcare comprises basic care, teaching (homeschooling), and recreational activities like talking, reading or playing with a child. Respondents had to answer these questions after having reported their time use for each of these subcategories. Thus, we assume that they were aware of the definition of housework and childcare when answering these questions. We define two of the main variables of interest based on these questions about the division of unpaid work within the household. First, we define the two dependent variables for the econometric analysis. These are dummy variables indicating whether the male partner took on more housework or childcare tasks during the lockdown than before the lockdown. These dummies are defined by combining the responses to HW.i, HW.ii, CC.i and CC.ii with information on the gender of the respondent. These dummy variables equal to one, if the value on the corresponding 11-point scale (zero to ten) was reported as being at least one point higher during the lockdown than before. 28% of all couples indicated that the male partner took on at least marginally more HW and 34% of all couples with children reported an increased involvement of the male partner in CC (see Table S1). Thus, we define the *change* in the division of HW and CC as an increased involvement of the male partner in these tasks. Second, we employ responses to questions HW.i and CC.i as measures for the division of housework and childcare prior to the lockdown. For this purpose, we subdivide the two 11-scale variables (indicating the pre-lockdown division of HW and CC) into four categories: "Woman does much more" (scale nos. 0–2), "Woman does more" (scale nos. 3–4), "Equal" (scale no. 5), "Man does (much) more" (scale nos. 6–10). Owing to the fact that in very few households men are primarily responsible for housework and/or childcare, we did not differentiate between "more" and "much more" in the case of males (see Table S1)."

RC7: The majority of responses come from women, have you used only those coming from the women as a robustness check i.e excluding those coming from men/both and compared responses when you have information from both partners? I imagine that there may be some disagreement on how does how much between partners (I have seen this in other data sets where division of labour is collected from both partners)

Response to RC7: The original supplementary document already included the results of a robustness test that addresses this question. In order to control for the gender of the survey respondent, we added a dummy variable indicating if the questionnaire was filled out by the male partner (see Table S6 "Additional control variable: information supplied by man" in the supporting material). In fact, this variable is highly significant for housework but not for childcare tasks. This might indicate there is some disagreement on the division of housework between men and women, but that there is only little disagreement in respect to the division of childcare. Moreover, the probability that men take on more housework is no longer significant for the whole sample. This is, however, no surprise as Table S4 already revealed that this effect is driven by households with children. All in all, the main results do not change substantially if we control for the gender of the survey respondent, but it once more indicates that gender norms are even more pronounced for childcare than for housework.

RC8: I found figures 1-3 quite difficult to interpret, I wonder if there are better ways to represent the descriptives, and draw out the key descriptive results?

Response to RC8: Thank you very much for pointing this out. It is very important that the readers can easily understand the visualization of our descriptive statistics. To achieve this, we revised figures 1-3. We are convinced that these now convey a better and clearer understanding of the descriptive results, and we have added an illustrative reading example below each figure included in the manuscript and in the supplementary document. We have changed the text accordingly in section 3 "Results" of the manuscript and in the supporting material. In particular figures 2 and 3 have a new outlook, which makes them even more straightforward to interpret. We will summarize the changes here for the sake of completeness and you can find the new figures attached at the end of this letter:

Figure 1 [FIG-1] (and Figure S1 [FIG-S1]): The radar charts display the average agreement of women and men (with kids in [FIG-1] and without kids in the supporting info in [FIG-S1]) to different statements on WFH. In FIG-1A the respondents were asked whether they 'Strongly agree', 'Rather agree', 'Rather disagree' or 'Strongly disagree' to statements on (dis-)advantages of WFH, and FIG-1B displays statements on the quality of WFH. The center of this spider web would indicate strong disagreement, the outer edges strong agreement. Put differently, the bigger the distance on the axis to the centre, the more the respondents agree on average with the statement. Blue triangles represent (averaged) answers from women, pink circles represent men's responses. For example, regarding the first statement of FIG-1A on whether the respondents agree with a good 'compatibility of free time and career while WFH' we see, that the pink circle is further away from the axis center, than the blue triangle. This reveals, that on average men (pink) found the statement to be more true (they stronger agree) than women (blue). The statements are arranged such that the reader sees the biggest differences between men and women (in average answers given) at the top of the spiderwebs, whereas in clockwise rotation we see the differences converging to same levels of (dis-)agreement, i.e. for the statement of 'good contact to boss' for example.

Figure 2 [FIG-2]: We summarized the formerly two separate pie charts into one and simplified the answer categories from six to four. The main message stays the same, as it reveals that women more often had to supervise the children while working from home (ca. 40%) as compared to men (roughly 25%).

Figure 3 [FIG-3]: Instead of a stacked bar plot, we constructed a histogram where the differently coloured categories of couples are visible next to each other for each value of the common x-axis. The x-axis shows the division of unpaid work before the lockdown on the 11-point scale, once for unpaid work as in housework [FIG-3A] and once for childcare [FIG-3B]. The edges of the x-axis illustrate extreme points (no. zero indicating the woman did everything, no. ten indicating the man did everything before the lockdown), in the middle of the axis unpaid work is split equally between partners. The height of the bars indicate the number of couples identified with each scale number, divided into three groups. Each (coloured) group refers to *changes* of the division of unpaid work within couples during the lockdown, i.e. whether "the woman does more" (green bar), "nothing changed" (blue bar) or "the man does more" (grey-purple bar). Regarding for example scale no. 5 for housework (see FIG-3A): before the pandemic 152 couples shared housework equally. For 92 of these couples nothing changed during the lockdown (blue bar). In 38 couples the female partner took on a larger share compared to before (green bar), whereas the opposite (male partner took on a larger share) was indicated by the remaining 24 couples (grey-purple bar).
Figure S3 [FIG-S3] in the supporting material compares the overall distributions, neglecting the specific changes within couples per scale number. This is useful, as we can see that even though the overall distribution did not change too much, the within couple changes in division of unpaid work still matter.

RC9: I would like the discussion of the results to consider more of the magnitude of results and to have a greater understanding of how these results fit into the gender bargaining and division of labour literature. I think the initial discussion is there but could benefit from reference to more literature and theory.

Response to RC9: This is an important remark. Importantly, we now report the magnitude of the most relevant coefficients in the text, and we relate the quantities to each other. On top of that, we have included a discussion of the results in the context of the gender bargaining, division of labour and tasks specialization literature. Note that we find mixed evidence of bargaining effects in the division of housework, however, we are not able to confirm the presence of such an effect (as captured by the relative income of the household members) in the case of childcare. As we have incorporated a lot of changes in section 3.2 "Regression results", we refer to the marked version of the paper to see how we incorporated them in relation to RC9 and RC13, which also

refer to the results section.

RC10: Would it also help to use the age of the youngest child instead of the number of children as a robustness check - I wonder how important the age of children are in women doing more of the child care? Especially having very young children?.

Response to RC10: Thank you for this very helpful remark. We added an additional robustness test to the supporting material (see Table S11 “Alternative child variable: age youngest child” in the supporting material and Table 3 at the end of this letter) using the variable “age of the youngest child”. We measure the age of the youngest child living in a household by a categorical variable consisting of five different age groups: “0-2 years old”, “3-5 years old”, “6-9 years old”, “10-14 years old” and “no child under 15 years of age”. We define the age group “0-2 years” as the reference group. The results of this analysis are very similar to our main regression results in the paper: In model (1) and (2) of this robustness test, we find no significant effect on any age group coefficient (see Table S11). Similarly, we only find weakly significant and positive effects for number of children between 6 and 9 years of age in models (1) and (2) in the manuscript (see Table 1). In model (3) of the robustness test, we find a significant and negative effect of men taking on more childcare tasks during lockdown if the age of the youngest child is between 10 and 14 years (in comparison to partners with children between 0 and 2 years of age). This result is in line with the regression results in the manuscript as well. One possible explanation we discuss in the manuscript is, that older children might be more likely to manage the additional workload (e.g. home schooling) themselves. As mentioned in the paper, we think that the absence of a clear and significant pattern in this regard, points to the importance of persisting gender norms during the lockdown. Furthermore, we also control for pre-lockdown division of unpaid work, which probably depends on the children’s age. Overall, we are able to conclude that a different specification of the child(ren)’s age living in a household does not alter the results.

RC11: I wonder if grocery shopping was appealing as it was an excuse to get out of the house!

Response to RC11: Thank you for this interesting question. Yes, indeed, this is true, and has been found in other research that we cite. We added the sentence ”It is also conceivable that grocery shopping was welcomed by men to spend some time alone outside of their home.” in the discussion of the regression results in section 3.2.

RC12: Note the links to the supplementary material did not work for me so I could not see this.

Response to RC12: Thank you for letting us know. This is very unfortunate, since this document contains a lot of additional information on the sample, additional descriptive statistics, and a large number of robustness checks addressing the model specification. However, the manuscript as such should be clear and understandable without having to take a look at the supplementary material. Overall, we are convinced the manuscript has improved substantially due to the revisions you suggested. Nevertheless, we hope that you are able to access the document now, as it gives a better and broader understanding of the present work and findings.

RC13: I found it quite hard to get to the key results so the authors may want to consider how to restructure the results and discussion to guide the reader to the key findings.

Response to RC13: Thank you for this suggestion. We have discussed and tried several options, such as dividing the section into separate results and discussion sections. However, we have opted for another version. First of all, we included a paragraph that should guide the reader through the section at the beginning of section 3.2 “Regression results”. Second, we summarise the main findings at the beginning of the corresponding paragraphs in this section. Overall, we hope that this has improved the readability of the paper substantially. It should now be clear where to find the key results. Please note that we have made many changes to the results section, such that we refer to the marked version of the manuscript in order to easily identify the revisions.

Comments provided by the editor (EC)

EC1: Since your rely on multiple methods to collect interview data (mailing list, Twitter, Facebook), how does that impact your response rate and sample balance? In Austria, is there any gender difference in Twitter and Facebook usage? If so, could that affect gender balance in your original sample (N=2,113)? Please add a table in providing the breakdown of data by medium of interview/response and for each.

Response to EC1: Thank you very much for this complex and interesting question. In case there is a systematic difference in accessing the survey by medium (Twitter, Facebook, Mailing list) and other factors (such as gender), the balance of the sample and the results could be significantly biased indeed. However, looking at the information we have on the medium via which respondents entered the sample, we do not conclude that this is the case. The following table shows the medium type via which respondents entered the survey platform (Reference URL) by gender and selected household types.

Table 1. Reference URL by gender and selected household types

Channel	% Women (no. 1,617)	% Men (no. 464)	% Couples wo. kids (no. 594)	% Couples w. kids (no. 645)	% Singles (no. 344)
(a) Facebook	27 (552)	3 (67)	7 (141)	13 (261)	3 (59)
(b) Mailing/newsletter	6 (120)	3 (54)	2 (46)	2 (45)	2 (39)
(c) Twitter	4 (76)	2 (46)	1 (28)	1 (30)	2 (36)
(d) Web addresses	3 (62)	1 (14)	1 (30)	1 (13)	1 (11)
(e) No info	39 (805)	14 (282)	17 (349)	14 (296)	10 (199)

The total sample of N=2,081[1] individuals consists of persons who completed the entire questionnaire. It is unbalanced in terms of gender, such as the sub sample used for the regression analysis (1,116 individuals/558 couples): Among the 2,081 respondents, 77% are women (1,617), and 23% (464) are men. The snowball sampling strategy therefore has appealed disproportionately to female respondents. Importantly, this is irrespective of the specific channel used by the respondents (mailing lists, Facebook, Twitter. etc.). Table 1 shows the percentage and number of respondents by each of the 5 aggregated data collection groups: (a) Facebook, (b) mailing lists/newsletters, (c) Twitter, (d) (various) web addresses or (e) no information. Group (e) is, by far, the largest group. We have no information how these respondents retrieved/ reached the survey. Facebook (a) and mailing lists/newsletters (b) were the most effective channels. As stated above, the majority of responses is coming from women, but this is not depending on the channel in a clear and distinct way. In Austria, Facebook ranks first place in the social media users (5,4 Mio.), whereas the number of Twitter users is still relatively small (0,16 Mio.).[2] 49,6% of all Facebook users in Austria are female (50,4% are men). Unfortunately, we could not find official data on Twitter usage by gender.

To conclude, we are confident in stating that the data collection method *ex ante* did not lead to an unbalanced sample. However, certain factors influenced the response rate of our targeted individuals (irrespective of the media channel). First, since the survey was voluntary (and lengthy), and the covered topics (unpaid work, time use during lockdown, work from home, mental health, etc.) seem to have been of greater interest to women than to men. Second, the time to implement, finalize and distribute the questionnaire had to be realized within a few weeks on the basis of small funds. This has prevented us from data collection via external opinion poll and survey institutes (both by time and budget constraints). But we do acknowledge that working with such institutes may have led to a more balanced and representative sample. Nevertheless, as or main interest lies is working couples, we focus on a specific part of the population that we reached relatively well. Third, distributing the survey *online* implies that respondents must have some sort of internet access (via smartphone, tablet or their personal computer) in order to participate in the study. We are well aware that this is an additional limit, in particular in terms of the coverage of older generations. Connected to this presupposition, in particular the distribution via mailing lists, Facebook or Twitter targets working people who use their computer or laptop (working from home during the lockdown) for work, rather than employees in critical infrastructure (i.e. supermarkets, hospitals, etc.). Against this background, we disseminated the survey broadly and via different channels.

[1]Again, we have restricted the total sample to individuals who identify themselves as women or men to break up the statistics by sex (rather than gender), thereby excluding roughly 30 non-binary respondents.

[2]Please refer to **statista.com** for further information.

EC2: Equally, is there similar difference by marital status? If so, did that affect the mix of single individuals and heterosexual couples in your original sample? I also wonder how your data collection method affects your answers given that individuals WfH would be more respective to digital communication on and if so whether that inflates the proportion WfH in your data (owing to sample selection bias, since on page 8 you admit the WfH wasn't universal despite govt regulation)? Again, I am sympathetic to "snowball sampling design" during COVID times. But we'd be open and frank about all the limitations including any systematic bias in sample composition and wherever possible, control for it in regression analysis (akin to "enumerator fixed effects" in face-to-face survey). Alternatively just acknowledge in your data limitation section.

Response to EC2: This comment raises a similar and interesting question: could we have handled the data collection differently to avoid a unrepresentative sample, and how does the sampling strategy affect the results? Thank you for stressing this point again, and we totally agree that a "snowball sampling strategy" poses specific challenges and limitations that have to be communicated openly. As argued in the response to EC1, we are confident in stating the sampling strategy as such did not cause an unbalanced sample. However, we have been very open and frank about the skewed sample in the data description (section 2), in the discussion of the results (section 3) as well as in the limitations (section 4) and concluding remarks (section 5). Especially regarding the regression outcomes we do not think that our subsample is unsuitable. Here, we want to explain more precisely why: The total sample comprises different household types, but respondents were not asked about their marital status. We hence are not able to study whether marital status *per se* had an influence on the response rate. Instead, the respondents had to report their relation to other members of the household, individually for each other person living in the household (i.e. if the reference person lives with a partner, with kids, or other adults). In case respondents reported that they cohabit with their partner (irrespective of marital status), they had to answer a very detailed module providing information about their partner (i.e. the partner's age, income, employment status, etc.). Therefore, Table 1 also provides information on the response channels (Facebook, Twitter etc.) separately for individuals living in couple households with or without kids as opposed to single households. The patterns by household type do not to deviate from the overall trend, but keeping in mind that we do have "no info" about the response channel for most individuals, followed by Facebook and mailing lists.

As stated in the response to EC1, we acknowledge that the sampling method comes with certain presuppositions, i.e. an internet access and availability of a communication device for using social media. In the data description, the discussion of the results, and in the limitations section, we acknowledge that the sample we use is not representative of the Austrian population. We also recommend to interpret the results as a upper bound estimates, as compared to what can be expected for the total Austrian population.

Also, we conducted a large set of sensitivity tests, which we reference in the manuscript and include in the supplementary material. What is important in the context of this comment is the robustness test that introduces a dummy variable controlling for the gender of the respondent (variable is equal to '1' in case the respondent is male). The corresponding coefficient is highly significant and positive in case of the regression that explains the change in the division of housework. Hence, the estimated probability of men taking on more housework during the lockdown (as compared to before) is higher if the information was provided by a men. However, this is not the case in the regression that studies the change in the division of childcare. Most importantly, the coefficients on the other variables included in the model do not change significantly (in terms of statistical significance and magnitude). A more detailed description of this test can be found in section "A.ii Controlling for the gender of the survey respondent" on the robustness tests in the supplementary information document.

EC3: Please describe sample size more clearly. You claim to focus on "730 heterosexual couples (1,460 individuals)" but in table S1, it is adding up to 1377 (687+690) while in Table 1, it is 1159. Final sample size is expected to be stable across all results table. If you did not restrict analysis to a common sample (with non-missing cases), please revise all Tables accordingly.

Response to EC3: Thank you for this remark. We restricted the analysis to a common sample with full information on all covariates (558 heterosexual couples | 1,116 individuals). We recalculated all descriptive statistics and regressions based on this sample, and we have adjusted all relevant text passages and tables. Additionally, we had to exclude one observation from the sample used in the regressions of the previous version of the manuscript. For this observation, only information on the division of childcare was available, as the information on the division of housework is missing. This led to very minor changes in the results. Therefore, all regression tables are marked "red" to indicate that some numbers have changed (minimally). Importantly, none of these changes has had an effect on our conclusions.

Response to Journal Requirements

1. Please ensure that your manuscript meets PLOS ONE's style requirements, including those for file naming.

Response: The manuscript meets all style requirements. It is based on the template files. We also meet the requirements for file naming.

2. Acknowledgments Section: Move New Information to the Financial Disclosure: "Thank you for stating the following in the Acknowledgments Section of your manuscript: [copy in statement] We note that you have provided funding information that is not currently declared in your Funding Statement. However, funding information should not appear in the Acknowledgments section or other areas of your manuscript. We will only publish funding information present in the Funding Statement section of the online submission form. Please remove any funding-related text from the manuscript and let us know how you would like to update your Funding Statement. Currently, your Funding Statement reads as follows: Judith Derndorfer, Vanessa Lechinger, Katharina Mader and Eva Six thankfully acknowledge funding from the Vienna Science and Technology Fund (WWTF), grant number COV20-040, and the Chamber of Labour Vienna grant Multiple Burdens of COVID-19. The funders had no role in study design, data collection and analysis, decision to publish, or preparation of the manuscript

Response: We have not declared any funding information in the Acknowledgments section or other sections of the manuscript.

The Acknowledgments sections of the manuscript reads as follows:

"We thank numerous participants at various seminars and conferences, for instance, at the Vienna University of Economics and Business, the Research Institute Economics of Inequality, the Institute for Advanced Studies Vienna, the EFAS network, the Young Economists Conference 2020 and the Momentum Congress 2020 for their helpful comments and feedback on previous versions of this manuscript. In addition, we thank Wilfried Altzinger, Karin Heitzmann, Petra Sauer and Mathias Moser for comments on the manuscript. We thank Julia Hoffmann, Mathias Moser and Alyssa Schneebaum for commenting the questionnaire and the survey design."

The Funding Statement reads as follows:

"Judith Derndorfer, Vanessa Lechinger, Katharina Mader and Eva Six thankfully acknowledge funding from the Vienna Science and Technology Fund (WWTF), grant number COV20-040, and the Chamber of Labour Vienna grant *Multiple Burdens of COVID-19*. The funders had no role in study design, data collection and analysis, decision to publish, or preparation of the manuscript."

3. We note that you have stated that you will provide repository information for your data at acceptance. Should your manuscript be accepted for publication, we will hold it until you provide the relevant accession numbers or DOIs necessary to access your data. If you wish to make changes to your Data Availability statement, please describe these changes in your cover letter and we will update your Data Availability statement to reflect the information you provide.

Response: We do not wish to make changes to the Data Availability statement. The Data Availability statement reads as follows:

"The dataset is available to the public at the Open Science Framework via the project *Multiple Burdens of COVID-19*."

4. Your ethics statement should only appear in the Methods section of your manuscript. If your ethics statement is written in any section besides the Methods, please delete it from any other section.

Response: Thank you for pointing this out! We have removed the Ethics Statement from the manuscript and we now provide the corresponding information in the manuscript, section 2.2 (Data and Survey Design). This paragraph reads as follows:

"The present research conforms to the STROBE (Strengthening the reporting of observational studies in epidemi-

ology) reporting guidelines for case-control and cross-sectional studies. The research underlying the manuscript was conducted at the WU Vienna University of Economics and Business (www.wu.ac.at) and it conforms to the *Directive of the Rectorate for Research at Vienna University of Economics and Business on Responsible Research and Scientific Integrity*, it adheres to the guides for good academic practice of the Austrian Agency for Scientific Integrity and hence was approved by the Vice-Rector for Research of WU Vienna. Participation in the survey was voluntary. Before starting to answer the survey respondents had to give written consent that the resulting data will be used - only - for scientific research purposes by researchers. Respondents were informed that data will be reported only such that the identification of individual respondents will not be possible. Survey respondents had not to report any personal information that would enable the identification of single respondents, and hence the data is fully anonymous."

5. You can choose to upload a striking image in Editorial Manager when you submit your manuscript. The image must be derived from a figure or supporting information file from your submission. To upload a striking image use the drop down menu on the "Attach Files" page to select "Striking Image" then select the image you would like to represent your manuscript. The striking image will not appear in the PDF sent to reviewers and editors, so it is important to make sure all necessary figures for the review process are uploaded as separate "Figure" file types. Once your manuscript is accepted for publication, this image file will represent your article on the PLOS ONE homepage.

Response: We have uploaded a striking image.

6. Your striking image file will represent your article upon publication on the PLOS ONE homepage. The image must be derived from a figure or supporting information file from your manuscript. Ideally, striking images should be high resolution, eye-catching, single panel images that do no contain additional text, scale bars, or arrows. Please also keep in mind that PLOS's Creative Commons Attribution License applies to striking images. As such, please do not submit any figures or photos that have been previously copyrighted unless you have express written permission from the copyright holder to publish under the CCAL license. You can read more about PLOS's Creative Commons License on our homepage: http://journals.plos.org/plosone/s/licenses-and-copyright

Response: We have uploaded a striking image.

Figures and tables referenced in the responses to the comments

Figure 1. Average agreement with statements on WFH from couple households with children

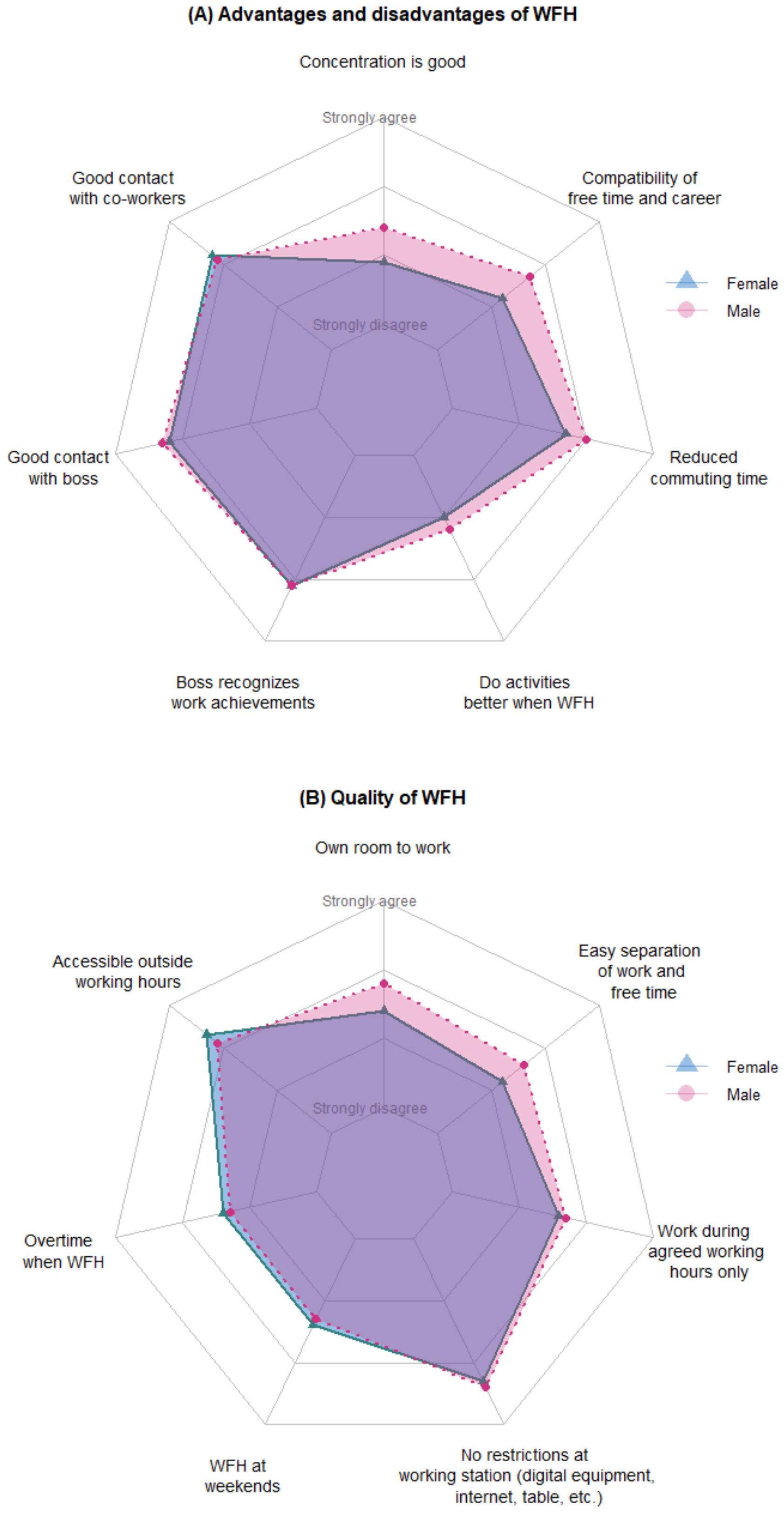

Figure 2. Main childcare arrangement during working hours by gender

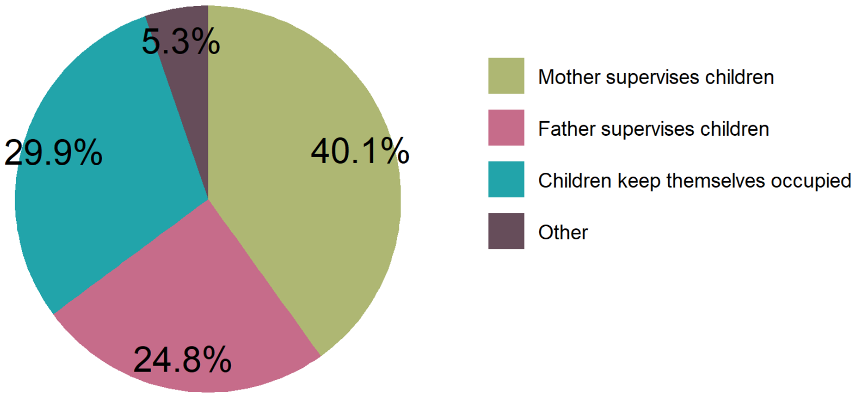

Figure 3. Division of housework (A) and childcare (B) before COVID-19 and subsequent changes during lockdown

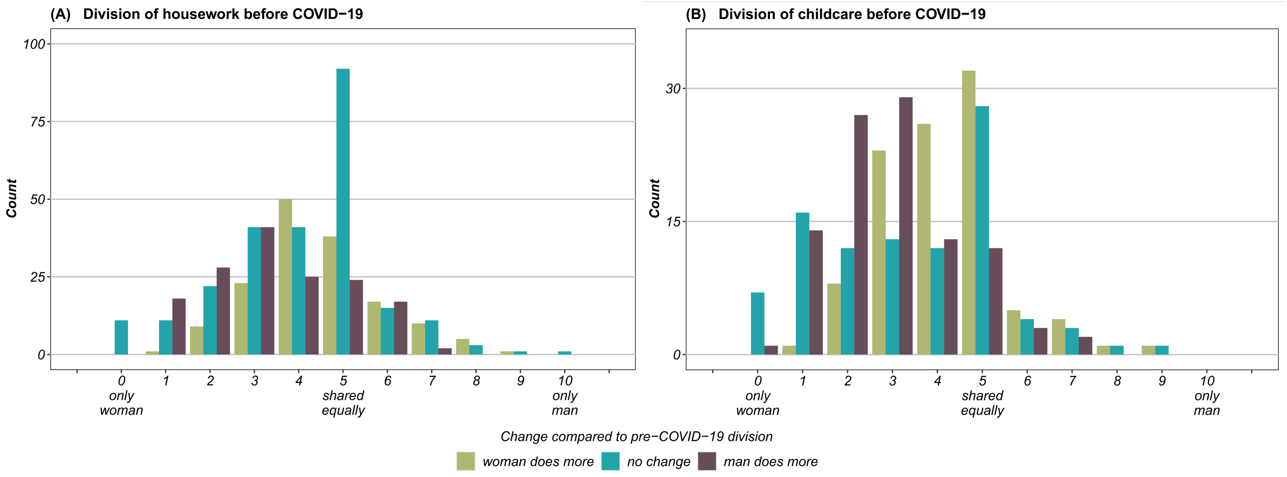

Table 2. Average time spent per activity during the lockdown by gender

	Male (N=550)			*Female (N=556)*		
	∅[a]	Participation rate[b]	∅ participants[c]	∅[a]	Participation rate[b]	∅ participants[c]
Activity	hh:mm	%	hh:mm	hh:mm	%	hh:mm
Paid work	07:48	97.6	08:00	06:44	98.7	06:49
Housework						
Cooking, baking, grocery shopping	00:52	75.8	01:09	01:29	95.7	01:33
Cleaning, laundry	00:41	75.5	00:55	01:09	93.5	01:14
Other: pet care, gardening, repairs	00:37	55.5	01:07	00:29	55.9	00:52
Childcare						
Physical care: feeding, washing, supervision	00:39	44.2	01:29	00:55	50.2	01:50
Learning, teaching	00:18	23.6	01:15	00:37	35.3	01:44
Leisure time: reading, playing, speaking with child	00:55	49.3	01:51	01:18	53.4	02:27
Personal care						
Sleeping	07:13	100.0	07:13	07:13	100.0	07:13
Eating, drinking, washing, breaks	01:44	97.5	01:47	01:42	96.8	01:45
Leisure time						
Sports, hobbies, media use	02:00	83.5	02:23	01:13	74.6	01:38
Social contacts	00:46	72.4	01:03	00:46	81.7	00:57
Voluntary work						
Helping high-risk group	00:05	9.8	00:52	00:07	15.5	00:46
Other: Red Cross, etc.	00:03	3.5	01:36	00:02	3.6	00:42
Other activity: Not specified	00:18	20.9	01:27	00:16	21.2	01:14
Total						
Housework	02:11	92.5	02:21	03:07	98.9	03:09
Childcare	01:52	56.7	03:17	02:50	60.3	04:43
Unpaid work	04:03	97.6	04:09	05:58	99.3	06:00
Paid and unpaid work	11:51	100.0	11:50	12:41	100.0	12:41

Note: Estimates by self and by partner are taken into account. If both partners filled out the survey, only the self-reported estimates are used.

[a]Mean time spent by all individuals.

[b]Share of individuals who spent some time on the activity.

[c]Mean time of all individuals who spent some time on the activity.

Table 3. Alternative child variable: age youngest child

	Dependent variable:		
	more HW: ♂ (1)	more HW: ♂ (2)	more CC: ♂ (3)
WFH: both	0.15 (0.07)**	0.19 (0.10)*	0.12 (0.10)
WFH: only ♀	0.11 (0.10)	0.06 (0.15)	−0.03 (0.12)
WFH: only ♂	0.22 (0.12)*	0.41 (0.14)***	0.32 (0.12)**
WFH: nobody (= *ref*)			
HW before: ♀ more	0.17 (0.05)***	0.15 (0.06)**	
HW before: ♀ much more	0.33 (0.06)***	0.34 (0.08)***	
HW before: ♂ (much) more	0.07 (0.07)	0.16 (0.11)	
HW before: equal (= *ref*)			
CC before: ♀ more			0.23 (0.06)***
CC before: ♀ much more			0.36 (0.07)***
CC before: ♂ (much) more			0.04 (0.12)
CC before: equal (= *ref*)			
Higher income: ♀	0.17 (0.07)**	0.19 (0.11)*	−0.01 (0.10)
Higher income: ♂	0.09 (0.04)**	0.10 (0.06)*	0.04 (0.06)
Equal income (= *ref*)			
Working hours ⩽20h: ♀	0.01 (0.05)	−0.01 (0.06)	−0.11 (0.06)*
Working hours ⩽20h (ST): ♀	−0.03 (0.07)	−0.02 (0.09)	−0.06 (0.09)
Working hours >20h (ST): ♀	−0.11 (0.16)	−0.15 (0.14)	0.06 (0.22)
Working hours >20h: ♀ (= *ref*)			
Working hours ⩽20h: ♂	0.16 (0.10)	0.34 (0.13)**	0.47 (0.09)***
Working hours ⩽20h (ST): ♂	0.08 (0.09)	0.05 (0.10)	0.14 (0.11)
Working hours >20h (ST): ♂	0.01 (0.10)	−0.20 (0.09)**	−0.28 (0.08)***
Working hours >20h: ♂ (= *ref*)			
Self-employed: ♀	−0.06 (0.06)	−0.09 (0.08)	−0.19 (0.08)**
Employed: ♀ (= *ref*)			
Self-employed: ♂	−0.07 (0.05)	−0.05 (0.07)	−0.19 (0.07)***
Employed: ♂ (= *ref*)			
Age youngest child: 3 – 5 years	0.02 (0.07)	−0.01 (0.07)	0.01 (0.07)
Age youngest child: 6 – 9 years	0.09 (0.08)	0.04 (0.08)	−0.07 (0.08)
Age youngest child: 10 – 14 years	0.02 (0.08)	−0.12 (0.09)	−0.24 (0.07)***
Age youngest child: No child < 15 years	0.08 (0.06)		
Age youngest child: 0 – 2 years (= *ref*)			
Age: ♀	−0.01 (0.00)	0.01 (0.01)	0.02 (0.01)**
Age: ♂	0.00 (0.00)	0.00 (0.01)	0.00 (0.01)
Educ. ♀ : Higher sec.	0.03 (0.06)	−0.00 (0.08)	0.09 (0.09)
Educ. ♀ : Lower sec. | prim.	−0.05 (0.07)	−0.01 (0.10)	0.17 (0.11)
Educ. ♀ : Tertiary (= *ref*)			
Educ. ♂ : Higher sec.	0.02 (0.05)	−0.00 (0.06)	0.07 (0.07)
Educ. ♂ : Lower sec. | prim.	0.00 (0.06)	−0.00 (0.09)	−0.07 (0.09)
Educ. ♂ : Tertiary (= *ref*)			
Observations	558	299	299
Log Likelihood	−301.70	−151.29	−154.90
Deviance	603.40	302.59	309.81
AIC	657.40	354.59	361.81
BIC	774.16	450.80	458.02

***$p < 0.01$; **$p < 0.05$; *$p < 0.1$

Note: WFH=working from home; HW=housework; CC=childcare; ST=short-time
